# Flash Communication: (Ph_3_P)_2_N_2_—Aza-Wittig Reagent for Metal Carbonyls

**DOI:** 10.1021/acs.organomet.5c00473

**Published:** 2026-01-15

**Authors:** Chandan Nandi, Fabian Dankert, Olha Bereziuk, Bernd Morgenstern, Robert Weiss, Dominik Munz

**Affiliations:** † Coordination Chemistry, 9379 Saarland University, Campus C4.1, D-66123 Saarbrücken, Germany; ‡ Inorganic Solid-State Chemistry, Saarland University, Campus C4.1, D-66123 Saarbrücken, Germany; § 9171 Friedrich-Alexander-Universität (FAU) Erlangen-Nürnberg, Nikolaus-Fiebiger-Str. 10, 91058 Erlangen, Germany

## Abstract

Cr­(CO)_6_, Mo­(CO)_6_, W­(CO)_6_, and Fe­(CO)_5_ undergo an aza-Wittig reaction with triphenylphosphinazine [(Ph_3_P)_2_N_2_]. These reactions address exclusively one CO ligand, yielding (isocyanoimino)­triphenylphosphorane metal complexes [(CO)_*x*_MCN_2_(PPh_3_)] (**1**: M = Cr, *x* = 5; **2**: M = Mo, *x* = 5; **3**: M = W, *x* = 5, **4**: M = Fe, *x* = 4). We present selective CO ligand deoxygenation at a metal center by an aza-Wittig reagent in the broad context of metallaheterocumulene fragmentation for carbon atom transfer.

## Graphical abstract

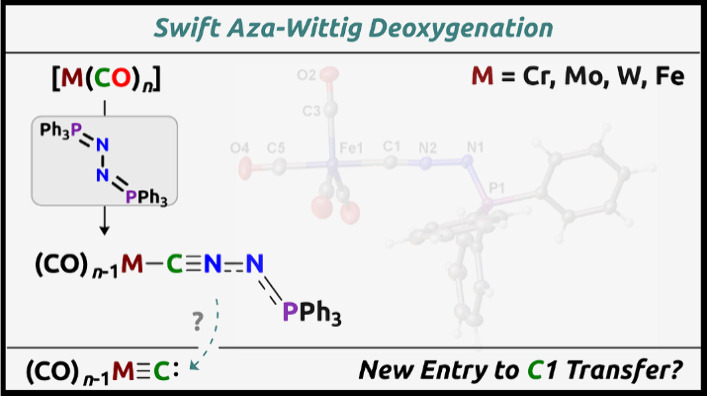

Fehlhammer and colleagues reported *N*-isocyanoiminotriphenylphosphorane (Ph_3_P=N_2_C; NIITP) in 1980.^1–4^ It emerged as a versatile CN_2_^2–^ transfer reagent in recent years, enabling the synthesis of, *inter alia*, oxadiazoles and diazoketenes.^5–7^ Just as is the case for Ph_3_P=CN_2_,^8^ it may also serve as a single-carbon atom transfer reagent under thermal conditions (Scheme 1a).^9,10^ Early to mid transition metal C_1_ surrogates (*i.e.*, terminal carbynes and carbides)^11^ can be synthesized via reduction of the corresponding metal carbonyls followed by O-acylation or silylation.^12–14^ Hayton et al. explored another approach, namely, the photochemical C atom transfer from Ph_3_P=N_2_C to cerium­(III), which however resulted in Ph_3_PN–NC bond cleavage without carbide formation.^15^ In search of the more reactive late transition metal and main-group metal congeners,^16^ we followed a related strategy harnessing metalla-heterocumulenes.^17–19^ These investigations showed that the fluoride-induced fragmentation affords a sufficient thermodynamic driving force to access an iron terminal nitride (Scheme 1b).^17^

**Scheme 1:**
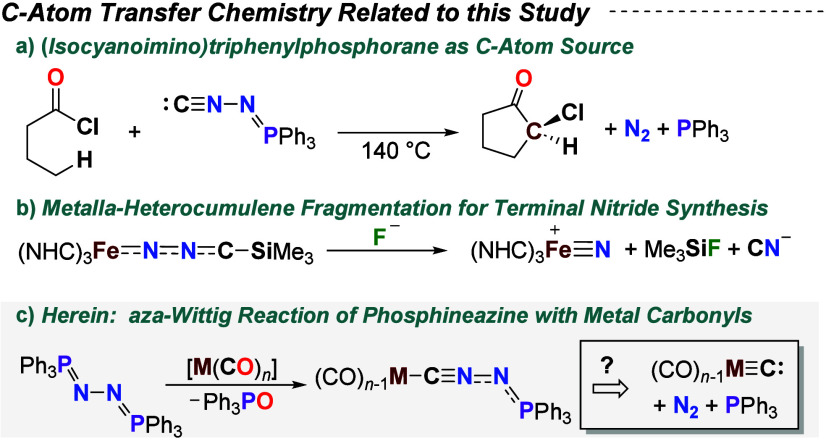
(a) *N*-Isocyanoiminotriphenylphosphorane as a C Atom Source; (b) Terminal Nitride Synthesis *via* Metalla-heterocumulene Fragmentation; (c) Phosphineazine for Generating a Metal Carbide Synthon *via* Aza-Wittig Deoxygenation of Metal Carbonyls

We hence hypothesized that the fragmentation of metal–*N*-isocyanoiminotriphenylphosphoranes could provide terminal carbides (Scheme 1c). However, accessing these complexes has hitherto been feasible only through ligand substitution.^1–3,20^ Whereas the Wittig reaction of carbon–phosphorus ylides (including carbadiphosphoranes) with metal carbonyls has been extensively studied since the first report by Kaska et al. in 1974,^21^ the imino-analogous aza-Wittig reaction^22,23^ is established only for converting organic carbonyls (R_2_C=O) to imines (R_2_C=NR′). However, it is known that treating Cr, Mo, W, Re, Os, and Ru carbonyls with Ph_3_P=NR′ may afford isocyanide complexes MCNR′.^24–30^ Further, corresponding N-heterocyclic carbene (NHC)^31–34^ complexes [M­(CO)_5_(NHC)] can be obtained by treating homoleptic metal carbonyls with the bifunctional iminophosphorane RNH­(CH_2_)_*n*_N=PPh_3_ (R = H, Et, Ph; *n* = 2, 3, 4).^28,35^ Herewith we report that Appel’s triphenylphosphineazine (Ph_3_P=N—N=PPh_3_)^36–39^ swiftly converts metal carbonyls to metal *N*-isocyanoiminotriphenylphosphoranes, hence providing an alternative synthesis of *N*-isocyanoiminotriphenylphosphorane complexes.

Stirring Cr­(CO)_6_, Mo­(CO)_6_, and W­(CO)_6_ suspended in benzene solutions of red triphenylphosphineazine afforded homogeneous yellow solutions within 4, 4.5, and 2 h, respectively (Scheme 2a; Figures S25–S27). In the case of liquid and pentacoordinate Fe­(CO)_5_, quantitative conversion occurred essentially instantaneously upon mixing.

**Scheme 2:**
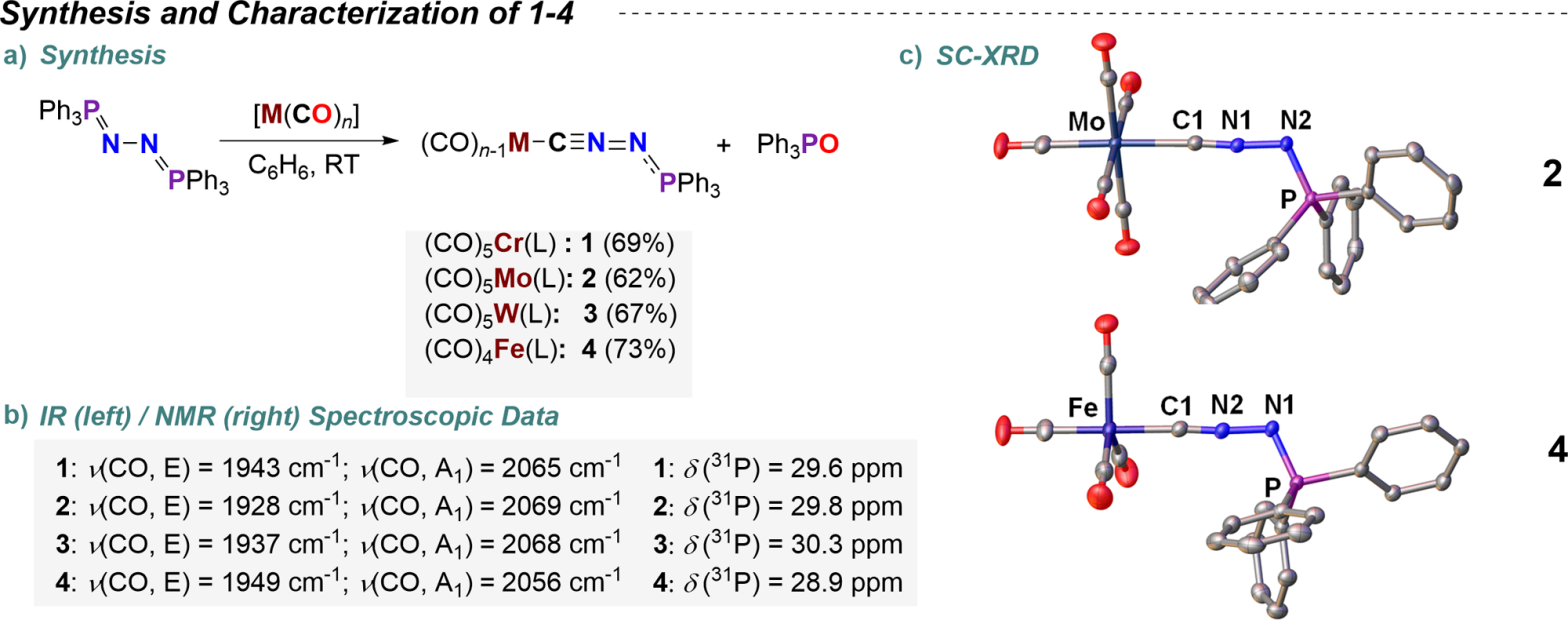
(a) Synthesis and (b, c) Selected Characterization Data^a^

a Thermal ellipsoids of X-ray structures are depicted at the 50% probability level. IR stretching modes were determined from neat substances. ^31^P NMR shifts were obtained from C_6_D_6_ solutions of isolated compounds.

The crude ^31^P­{^1^H} NMR spectra confirmed the quantitative formation of complexes **1**–**4** in stoichiometric ratios concomitant with triphenylphosphine oxide (Figures S1, S7, S13, and S19). The ^31^P­{^1^H} NMR shifts δ (**1**, 29.6 ppm; **2**, 29.8 ppm; **3**, 30.3 ppm; **4**, 28.9 ppm) are shifted toward low field with respect to the phosphineazine starting material (δ = 13.3 ppm),^38^ hence tentatively suggesting enhanced P=N ylide character. Running the reactions for **3** and **4** in more polar solvents (tetrahydrofuran (THF), *o*-difluorobenzene) with 2.5 equiv of homoleptic carbonyl precursor complex led to faster reactions (<10 min for **3**). Also in these solvents, **3** and **4** formed in quantitative crude yield without indication of a second aza-Wittig reaction with the second phosphonio substituent, even upon heating to 80 °C for 2 h (Figures S37–S42). Crystallization from *n*-pentane/*n*-hexane allowed removal of triphenylphosphine oxide, thereby affording analytically pure complexes **1**–**4** in isolated yields of 62–72%. The ^13^C­{^1^H} spectroscopic analysis confirmed the presence of the *C*N_2_ (**1**, 125.0 ppm; **2**, 125.5 ppm, **3**, 124.8 ppm, **4**, 125.0 ppm) and *C*O (see the Supporting Information) ligands, and the IR spectra of **1**–**4** display distinctive ν̃(CO, E) (**1**, 1943 cm^–1^; **2**, 1928 cm^–1^; **3**, 1937 cm^–1^; **4**, 1949 cm^–1^) and ν̃(CO, A_1_) (**1**, 2065 cm^–1^; **2**, 2069 cm^–1^; **3**, 2068 cm^–1^; **4**, 2056 cm^–1^) stretching bands. In accordance with the previous work by Fehlhammer,^2^ the ν̃(CN) bands are too weak to be discerned in the IR spectra (see Table S1 for further details).

Single-crystal X-ray diffraction (sc-XRD) studies allowed determination of the structural parameters in complexes **2**, **3**, and **4** (Scheme 2c, Table 1, and Figures S43–S45) and comparison with the values for **1**,^4^ originally synthesized by Fehlhammer et al. through treating (CO)_5_Cr­(THF) with *N*-isocyanoiminotriphenylphosphorane.^1–3,20^ The N—N bond lengths in complexes **1**–**4** range from 1.345 to 1.349 Å, which are significantly shorter than found for triphenylphosphineazine (1.497(2) Å), in a similar range as for the free ligand (1.345(4) Å), and consistent with a formal bond order of 1.5. The same is true for the N—P distances, which are moderately elongated (1.618–1.631 Å) with respect to Ph_3_P=N—N=PPh_3_ (1.582(1) Å). The nitrilimines’ C–N bond lengths (1.145 to 1.155 Å) are similar to those in the free ligand (1.153 Å) and typical for nitriles. In short, the combined vibrational-spectroscopic and structural data indicate delocalized π systems within the nitrilimine ligands and moderate activation via π back-bonding from the various metals.

**Table 1:** Structural Parameters of **1** ^4^ and **2**–**4** (*cf.* Figures S37–S39)

complex	*d*_M–C_ [Å]	*d*_C–N_ [Å]	*d*_N–N_ [Å]	*d*_N–P_ [Å]
**1** (M = Cr)	2.031(3)	1.151(4)	1.345(3)	1.618(2)
**2** (M = Mo)	2.181(1)	1.155(2)	1.345(2)	1.626(1)
**3** (M = W)	2.168(4)	1.145(5)	1.346(4)	1.620(3)
**4** (M = Fe)	1.911(2)	1.154(2)	1.349(2)	1.631(1)

To form the terminal carbides,^40^ complexes **1** and **3** were treated with the oxygen atom transfer (OAT) reagents Ag_2_O and iodosylbenzene. However, the NMR and IR spectroscopic analyses (Figures S29 and S32) revealed instead the formation of the homoleptic hexacarbonyl complexes and (CO)_5_M­(OPPh_3_). Also, treating **1** in *o*-difluorobenzene with tetramethylammonium or cesium fluoride did not lead to the selective abstraction of the phosphonio group even after refluxing at 100 °C (Figures S33–S36).

In closing, we report that Appel’s phosphineazine is a convenient reagent to synthesize *N*-isocyanoiminotriphenylphosphorane complexes via deoxygenation of transition metal carbonyls. The aza-Wittig reaction addresses exclusively one of the two triphenylphosphonio substituents. OAT reagents induce metallacumulene defragmentation and regenerate the homoleptic carbonyl complexes.

## Supplementary Material



## Notes

Synthetic procedures, spectroscopic data, crystallographic details (PDF)
