# Supplementary material for: Flash Communication: (Ph3P)2N2—Aza-Wittig Reagent for Metal Carbonyls
Source: Organometallics. 2026 Jan 15;45(3):249–52. doi: 10.1021/acs.organomet.5c00473 (PMC12892306; doi:10.1021/acs.organomet.5c00473)
Supplement: Supplementary file 1 [file om5c00473_si_001.pdf]

SUPPORTING INFORMATION

Flash Communication: $(Ph_3P)_2N_2$ – *aza*-Wittig Reagent for Metal Carbonyls

Chandan Nandi,[a] Fabian Dankert,[a] Olha Bereziuk,[a] Bernd Morgenstern,[b] Robert Weiss,*[c] Dominik Munz*[a]

[a] Saarland University, Coordination Chemistry, Campus C4.1, D-66123 Saarbrücken, Germany.

[b] Saarland University, Inorganic Solid-State Chemistry, Campus C4.1, D-66123 Saarbrücken, Germany.

[c] Friedrich-Alexander-Universität (FAU) Erlangen-Nürnberg, Nikolaus-Fiebiger-Str. 10, D-91058 Erlangen, Germany.

Email: weiss@chemie.uni-erlangen.de; dominik.munz@uni-saarland.de

Table of Contents

1 General Experimental Details S3

2 Synthetic Details S5

2.1 $(CO)_5CrCN_2PPh_3$: **1** S5

2.2 $(CO)_5MoCN_2PPh_3$: **2** S9

2.3 $(CO)_5WCN_2PPh_3$: **3** S13

2.4 $(CO)_4FeCN_2PPh_3$: **4** S17

3 Kinetic Studies S21

4 Experimental and Computed IR Data of **1**–**4** S25

5 Reaction of **1** and **3** with Iodosylbenzene S27

6 Reaction of **1** with [Me_4NF] S31

7 Attempts Toward bis-*aza*-Wittig Reaction S35

8 sc-XRD Structure Elucidation and Refinement S43

9 References S46

1 General Experimental Details

If not stated otherwise, all manipulations were carried out under oxygen- and moisture-free conditions under an inert atmosphere of dinitrogen using standard Schlenk techniques or an UNILab pro MBraun glovebox. Solvents were collected from a solvent-purification system (SPS), degassed by freeze-pump-thaw, and stored over 3 Å molecular sieves. Molecular sieves were washed with copious amounts of distilled water in an Erlenmeyer flask (3×) to remove traces of chloride. Subsequent activation was achieved by heating to 250 °C under high vacuum for at least 5 days. Benzene, pentane and hexane were stored over a mirror of potassium. All chemicals including $Cr(CO)_6$, $Mo(CO)_6$, $W(CO)_6$, $Fe(CO)_5$ and KHMDS were purchased from SigmaAldrich/Merck and used without purification. The triphenylphosphineazine, $N_2(PPh_3)_2$, was prepared by following a modified method of Appel and co-workers,[1] that is by deprotonation of $[Ph_3PN(H)N(H)PPh_3]Cl_2$ with 2 eq. of KHMDS.[2] All glassware was pre-dried in an oven at 150 °C overnight and flame-dried directly prior to usage. C_6D_6 was obtained dry and packaged under argon and was stored over a mirror of potassium. NMR samples were prepared inside the glovebox in NMR tubes equipped with gas-tight *J. Young* valves.

Melting points were determined using an Electrothermal IA9200 Programmable Digital Melting Point Apparatus

High resolution mass spectrometry (HRMS) HRMS-APPI spectra were recorded on a Quadrupole Linear Ion Trap (QqLIT), AB Sciex API 5500 QTRAP. Electrospray-ionization MS (ESI-MS) measurements were performed on a UHR-TOF Bruker Daltonik (Bremen, Germany) maXis plus, an ESI-quadrupole time-of-flight (qToF) mass spectrometer capable of a resolution of at least 60.000 FWHM.

FTIR spectra for solid samples were recorded using a Bruker Vertex 70 spectrometer (Bruker Optics, Ettlingen, Germany), from 4000 to 500 cm^{-1} on attenuated total reflectance (ATR) mode. IR spectra were obtained as an average of 16 scans with a resolution of 0.5 cm^{-1}. Relative intensities are reported according to the abbreviations: weak (=w), medium (=m), strong (=s).

NMR spectra spectra were recorded either on a Bruker Biospin Avance II+ 400 MHz WB or a Bruker Avance III HD 300 MHz spectrometer at a probe temperature of 298 K if not otherwise indicated. The chemical shifts δ are calculated in ppm; the solvent residual signals of incomplete deuterated solvent molecules were used as internal reference for the ^{1}H NMR spectra, and the respective carbon solvent signals for the ^{13}C

NMR spectra. $^{31}P\{^{1}H\}$ spectra of the crude reaction mixtures were recorded with a relaxation time D1= 30 s to allow for quantification.

CHNS Elemental analyses were carried out with an Elementar vario Micro Cube instrument. Elemental analyses for compounds **1**, **2**, and **4** were conducted with a crude solid having 1:1 mixture of complex and PPh_3O, whereas elemental analysis of **3** was conducted with the recrystallized solid.

2 Synthetic Details

2.1 $(CO)_5CrCN_2PPh_3$: 1

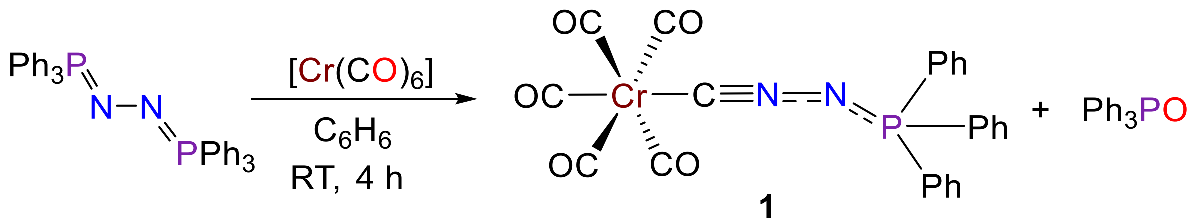

1

Inside the glovebox, a suspension of $[Cr(CO)_6]$ (26 mg, 0.12 mmol, 1.3 eq) was stirred in a glass vial with red triphenylphosphineazine $[(Ph_3P)_2N_2]$ (50 mg, 0.09 mmol, 1.0 eq) dissolved in 5 mL of benzene. The mixture was stirred for 4 h, upon which the color of the reaction mixture changed from red to yellow. The crude $^{31}P\{^1H\}$ NMR spectrum revealed the formation of $[(CO)_5CrCN_2PPh_3]$ and Ph_3PO in 1:1 ratio (Figure S1). The reaction mixture was then filtered, and the solvent was removed *in vacuo* to afford a 1:1 mixture of **1** and Ph_3PO. Triphenylphosphine oxide can be removed by extraction with pentane (2×10 mL), followed by cooling the solution to –35 °C overnight to afford $[(CO)_5CrCN_2PPh_3]$ (**1**)[3, 4] as pale-yellow crystals in 69% yield (28 mg, 0.056 mmol).

Note: If running the reaction in stoichiometric ratio, a homogeneous solution was obtained, yet we preferred to run the reaction with a slight excess of chromium hexacarbonyl due to the facile removal of this insoluble compound.

^{1}H NMR (C_6D_6, 400.1 MHz): δ = 7.49–7.44 (m, 6H, C*H*), 7.02–6.98 (m, 3H, C*H*), 6.96–6.91 (m, 6H, C*H*) ppm.

^{31}P NMR (C_6D_6, 162.0 MHz): δ = 29.6 ppm.

^{13}C NMR (C_6D_6, 100.6 MHz): δ = 219.4 (CO), 216.7 (CO), 133. 5 (d, $^4J_{CP}$ = 2 Hz, CH_{Ar}), 132.9 (d, $^3J_{CP}$ = 9 Hz, CH_{Ar}), 129.3 (d, $^2J_{CP}$ = 13 Hz, CH_{Ar}), 128.1 (d, $^1J_{CP}$ = 24 Hz, CH_{Ar}), 125.0 (d, $^1J_{Cr\text{–}C}$ = 97 Hz, $CrCN_2$) ppm.[5]

m.p.: 113 °C.

IR in THF: $\tilde{\nu}_{CO}$ = 1943 cm^{-1} (vs, E), 2065 cm^{-1} (w, A_1).

CHN for $C_{24}H_{15}N_2O_5PCr \cdot 0.2C_5H_{12}$ (isolated compound):

calcd. C 59.02, H 3.45, N 5.51;

found C 58.88, H 3.20, N 5.23.

HRMS (in THF, ESI$^+$): *m/z*: $[M+H]^+$ calcd. for $C_{24}H_{16}N_2O_5PCr$ 495.0201; found 495.0196.

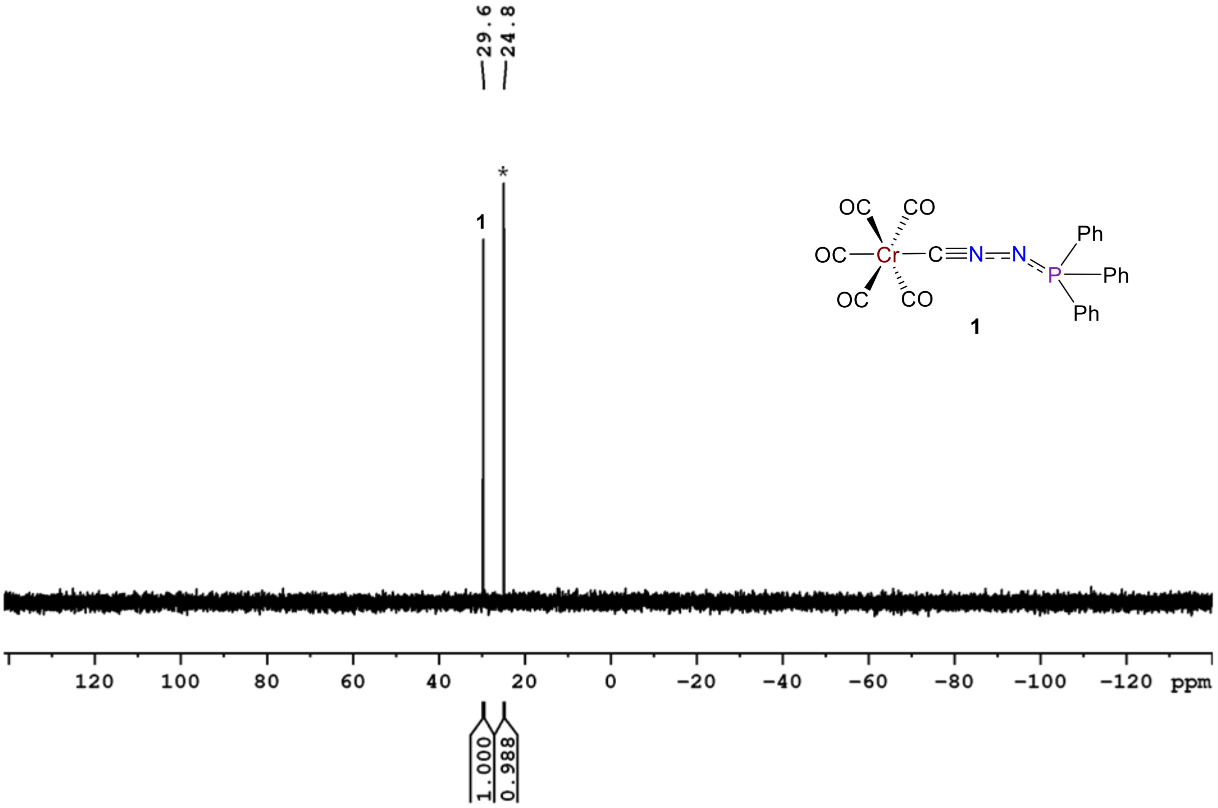

Figure S1. Crude $^{31}P\{^{1}H\}$ NMR spectrum obtained after stirring $[Cr(CO)_6]$ over a solution of $[(Ph_3P)_2N_2]$ for 3 h (C_6D_6, 162.0 MHz, D1 = 30 s, $*Ph_3PO$).

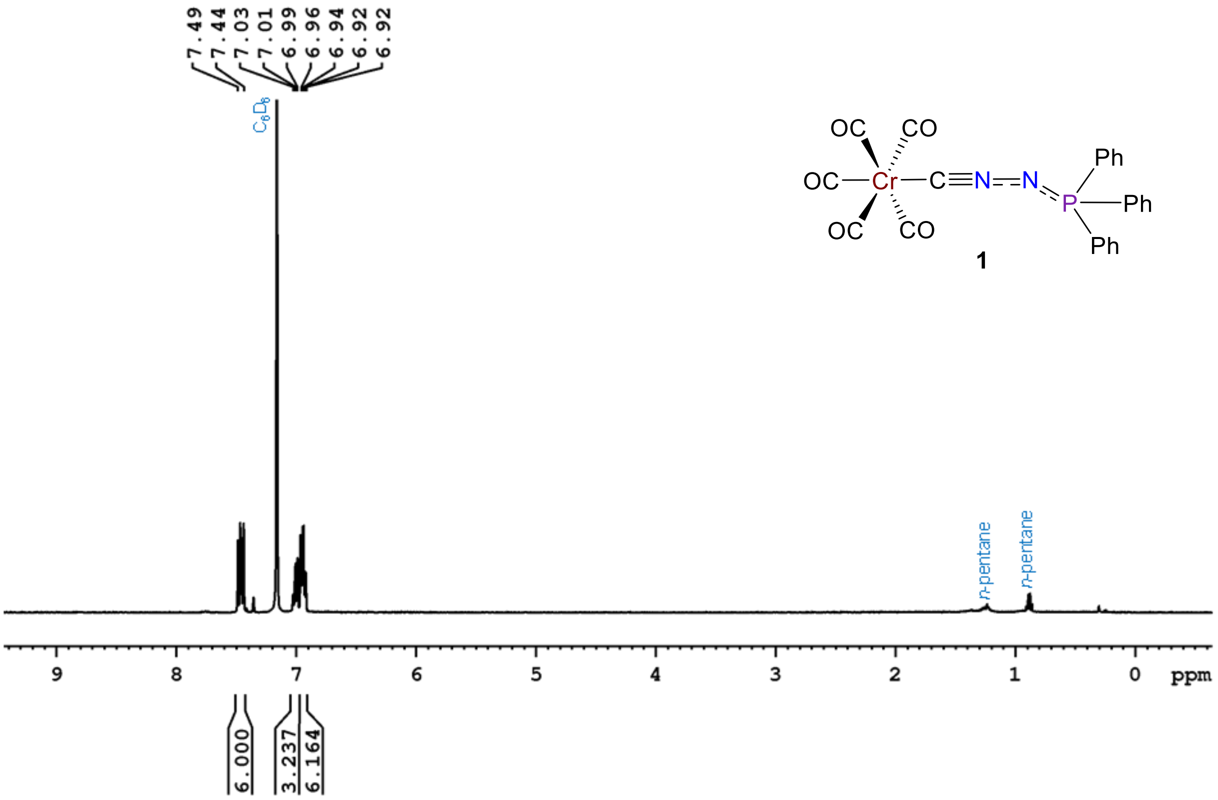

Figure S2. ^{1}H NMR spectrum of **1** (C_6D_6, 400.0 MHz).

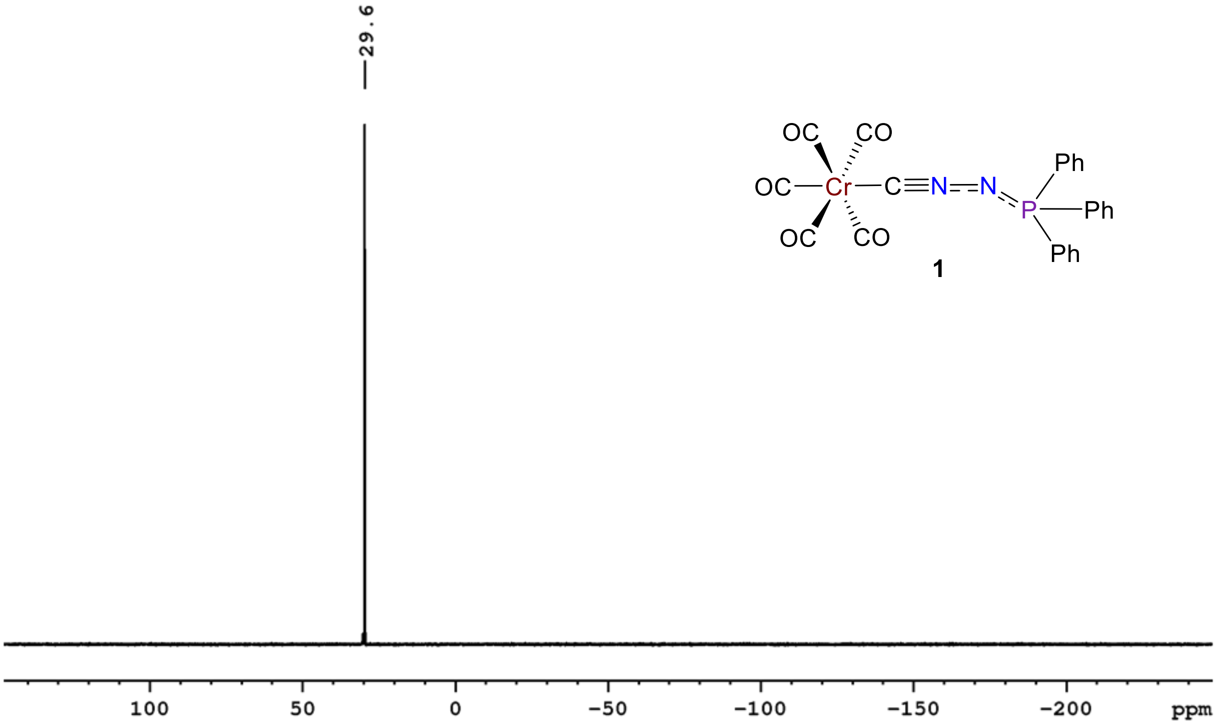

Figure S3. $^{31}P\{^{1}H\}$ NMR spectrum of **1** (C_6D_6, 162.0 MHz).

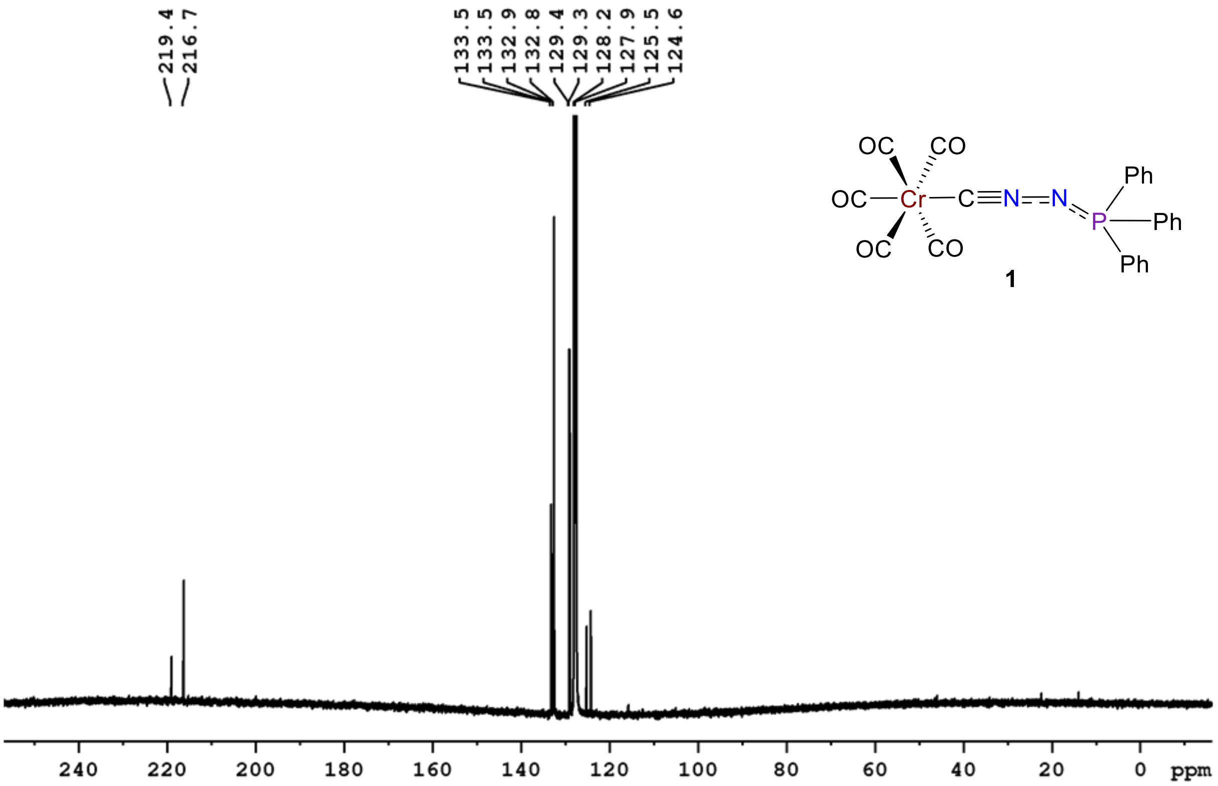

Figure S4. $^{13}C\{^{1}H\}$ NMR spectrum of **1** (C_6D_6, 100.6 MHz).

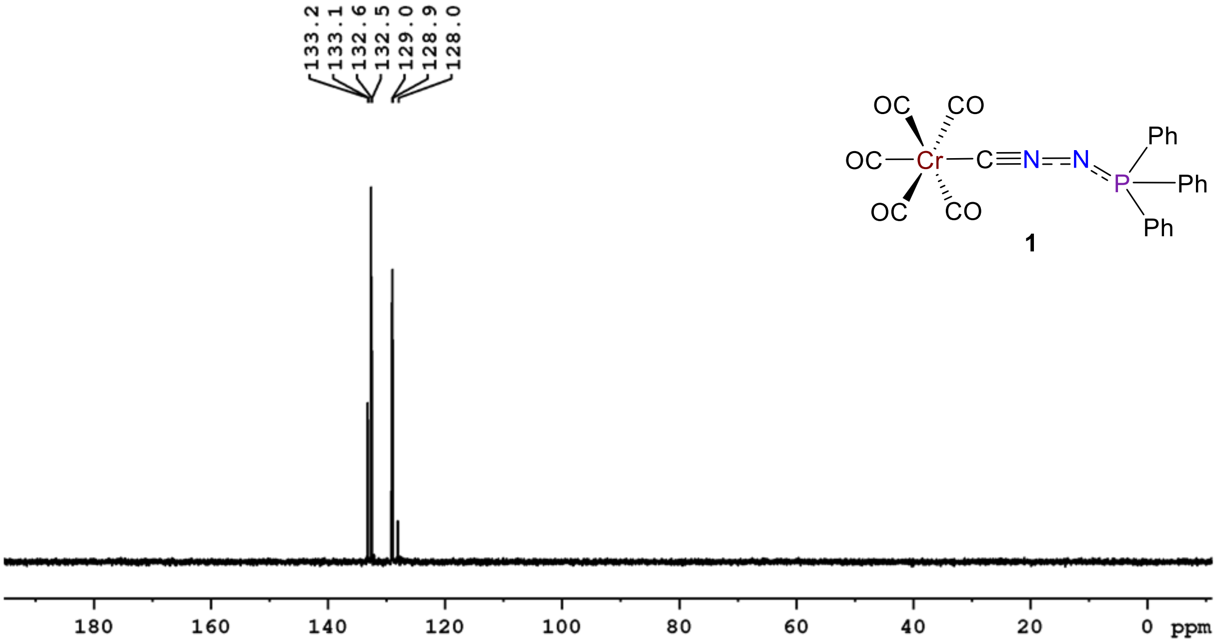

Figure S5. DEPT-135 NMR spectrum of **1** (C_6D_6, 100.6 MHz).

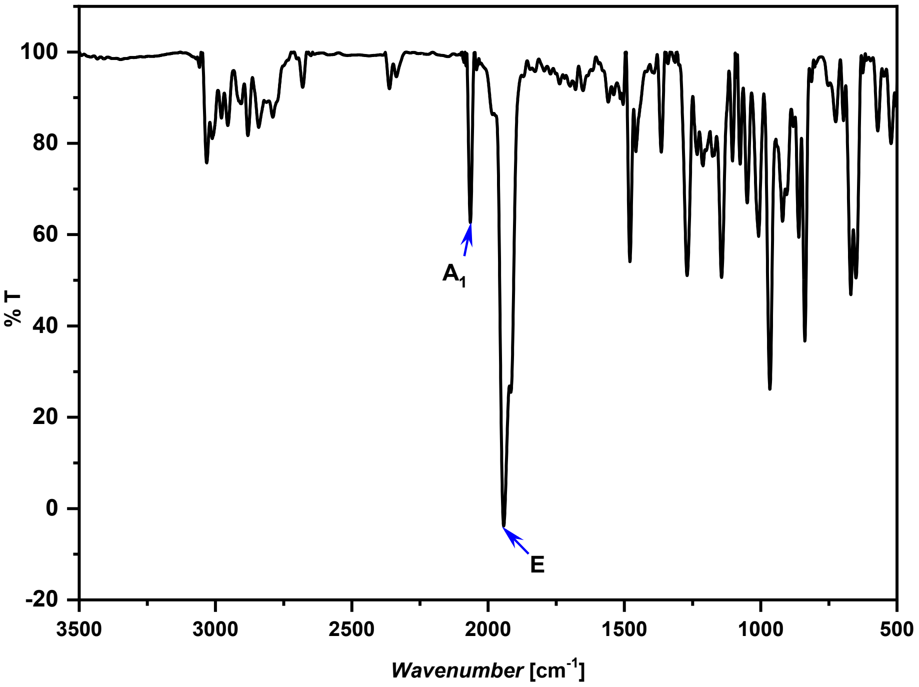

Figure S6. IR spectrum of **1** in THF.

2.2 $(CO)_5MoCN_2PPh_3$: 2

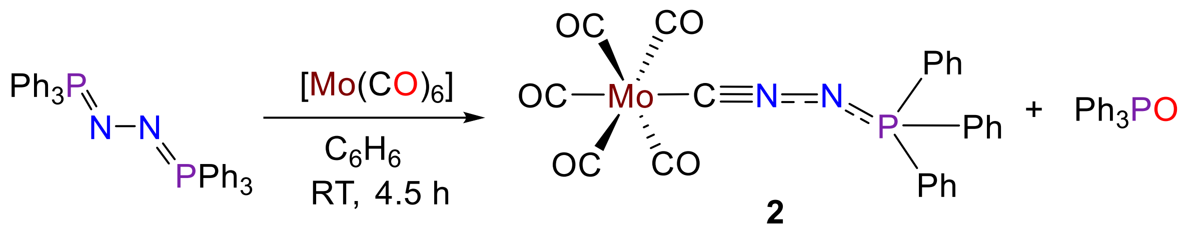

2

Inside the glovebox, a suspension of $[Mo(CO)_6]$ (19 mg, 0.07 mmol, 1.3 eq) was stirred in a glass vial with red triphenylphosphineazine $[(Ph_3P)_2N_2]$ (30 mg, 0.05 mmol, 1.0 eq) dissolved in 3 mL of benzene. The mixture was stirred for 4.5 h, upon which the color of the reaction mixture changed from red to yellow. The crude $^{31}P\{^1H\}$ NMR spectrum revealed the formation of $[(CO)_5MoCN_2PPh_3]$ and Ph_3PO in 1:1 ratio (Figure S7). The reaction mixture was then filtered, and the solvent was removed *in vacuo* to afford a 1:1 mixture of **2** and Ph_3PO (elemental analysis). Triphenylphosphine oxide can be removed through extraction by 10 mL of hexane, followed by cooling the solution to –35 °C overnight to afford $[(CO)_5MoCN_2PPh_3]$ (**2**) as pale-yellow crystals in 62% yield (18 mg, 0.054 mmol). These crystals are suitable sc-XRD.

Note: If running the reaction in stoichiometric ratio, a homogeneous solution was obtained, yet we preferred to run the reaction with a slight excess of molybdenum hexacarbonyl due to the facile removal of this insoluble compound.

^{1}H NMR (C_6D_6, 400.1 MHz): δ = 7.48–7.42 (m, 6H, C*H*), 7.02–6.99 (m, 3H, C*H*), 6.96–6.92 (m, 6H, C*H*) ppm.

^{31}P NMR (C_6D_6, 162.0 MHz): δ = 29.8 ppm.

^{13}C NMR (C_6D_6, 100.6 MHz): δ = 209.0 (*C*O), 205.3 (*C*O), 133. 2 (d, $^4J_{CP}$ = 3 Hz, CH_{Ar}), 132.9 (d, $^3J_{CP}$ = 9 Hz, CH_{Ar}), 129.3 (d, $^2J_{CP}$ = 12 Hz, CH_{Ar}), 128.1 (d, $^1J_{CP}$ = 24 Hz, CH_{Ar}), 125.5 (d, $^1J_{Mo\text{–}C}$ = 97 Hz, $MoCN_2$) ppm.[5]

m.p.: 110 °C.

IR in THF: $\tilde{\nu}_{CO}$ = 1929 cm^{-1} (vs, E), 2069 cm^{-1} (w, A_1).

CHN for $C_{24}H_{15}N_2O_5PMo \cdot 1.05\ C_{18}H_{15}PO \cdot 0.6C_6H_6$ (prior to hexane extraction):

calcd. C 63.09, H 4.38, N 3.21;

found C 62.76, H 4.77, N 2.80.

HRMS (in THF, ESI$^+$): *m/z*: $[M+H]^+$ calcd. for $C_{24}H_{16}N_2O_5PMo$ 540.9851; found 540.9864.

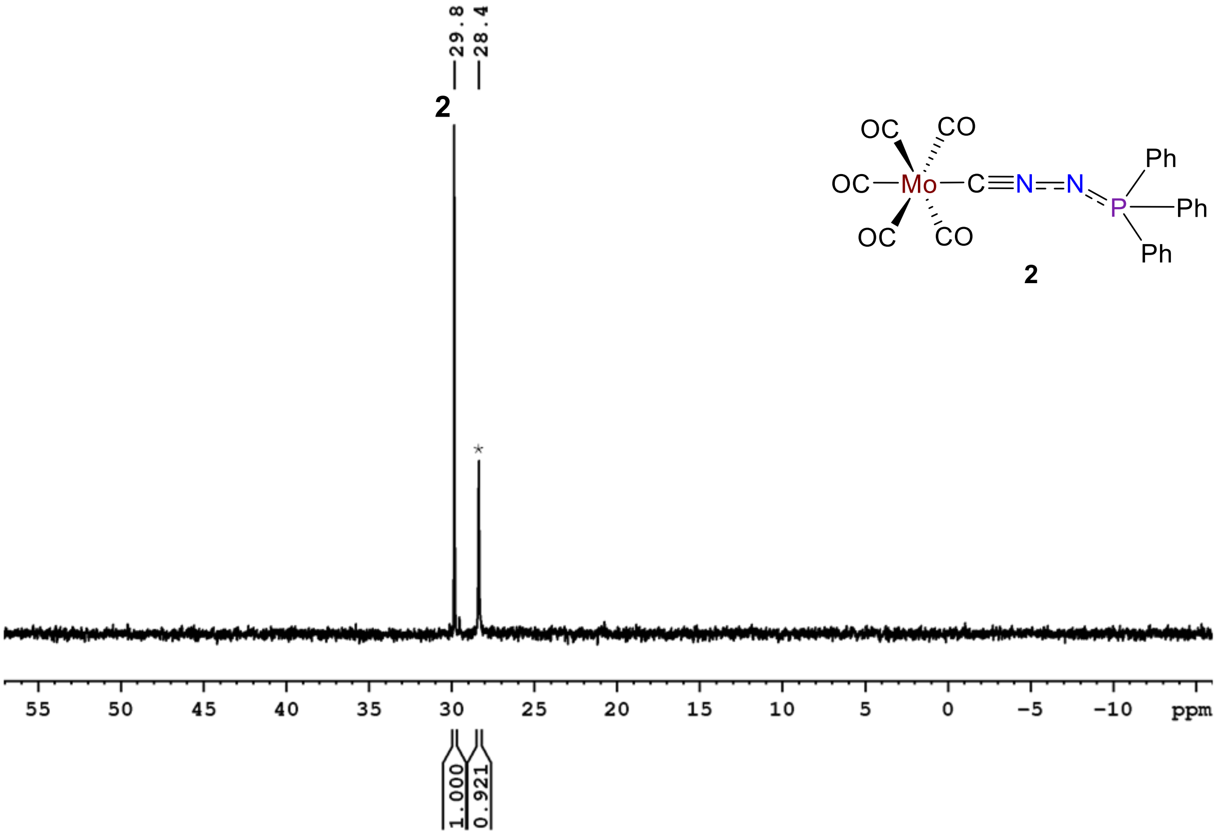

Figure S7. Crude $^{31}P\{^{1}H\}$ NMR spectrum obtained after stirring $[Mo(CO)_6]$ over a solution of $[(Ph_3P)_2N_2]$ for 4 h (C_6D_6, 162.0 MHz, D1 = 30 s, $*Ph_3PO$).

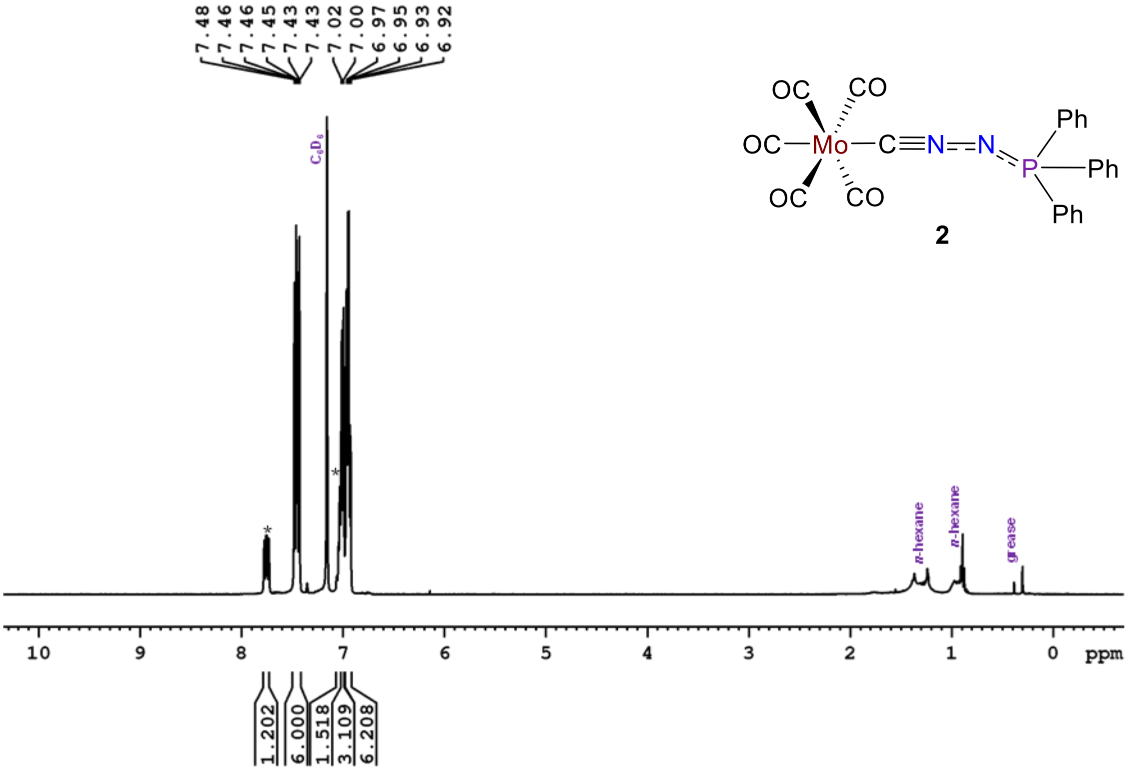

Figure S8. ^{1}H NMR spectrum of **2** (C_6D_6, 400.0 MHz, $*Ph_3PO$).

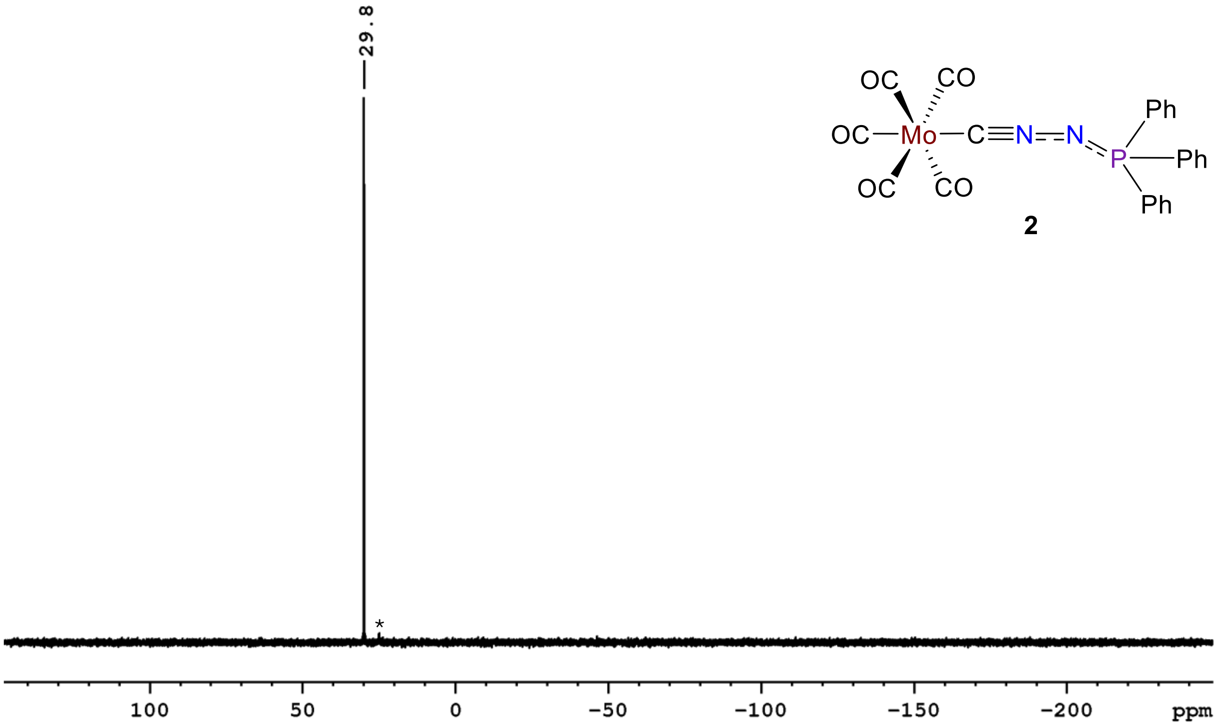

Figure S9. $^{31}P\{^{1}H\}$ NMR spectrum of **2** (C_6D_6, 162.0 MHz, *Ph_3PO).

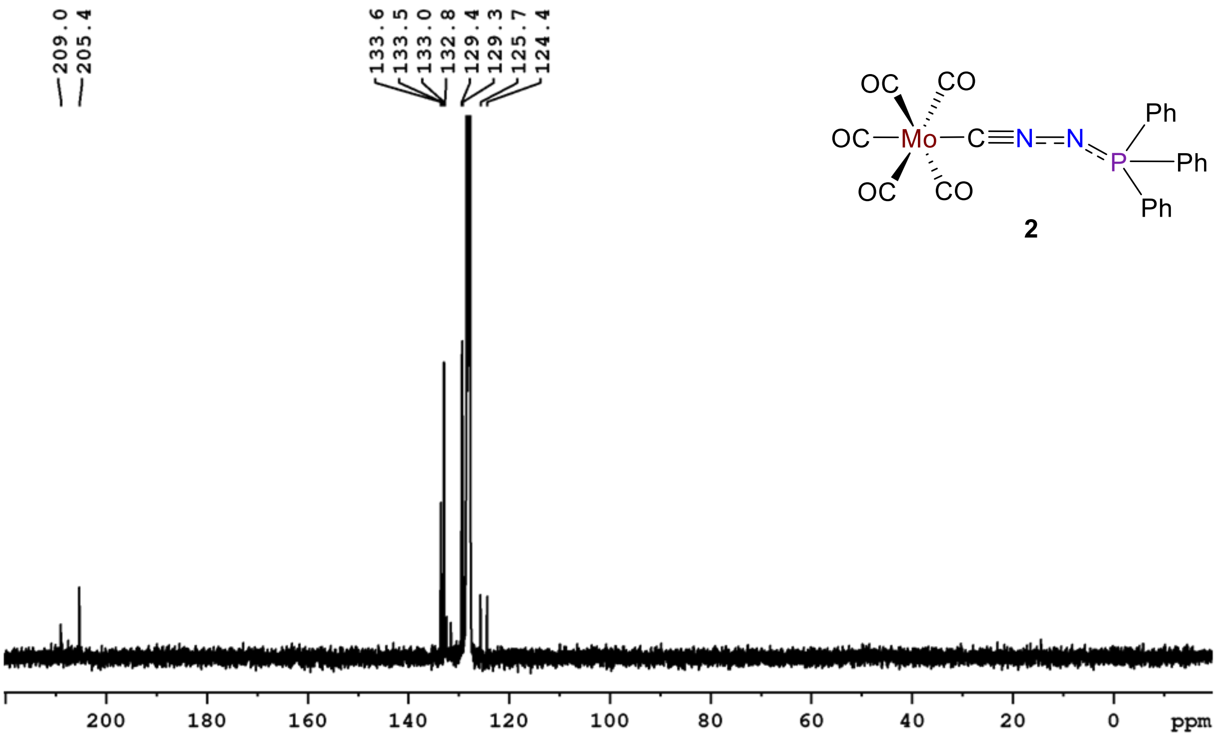

Figure S10. $^{13}C\{^{1}H\}$ NMR spectrum of **2** (C_6D_6, 100.6 MHz).

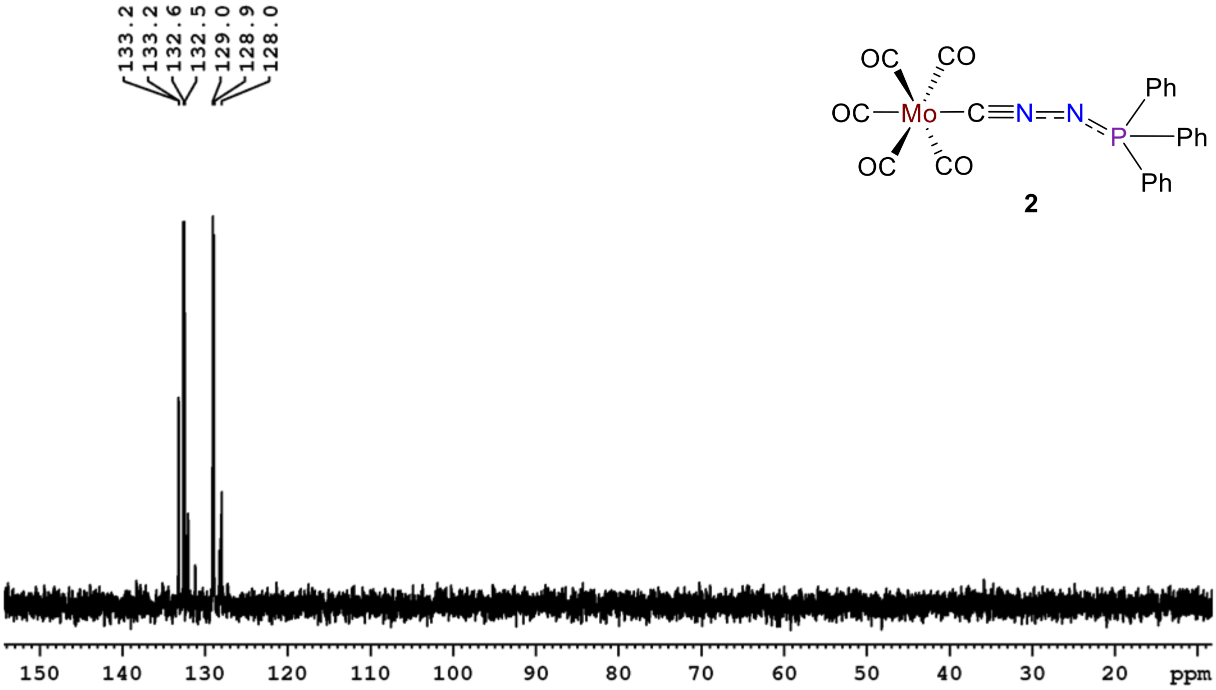

2

Figure S11. DEPT-135 NMR spectrum of **2** (C_6D_6, 100.6 MHz).

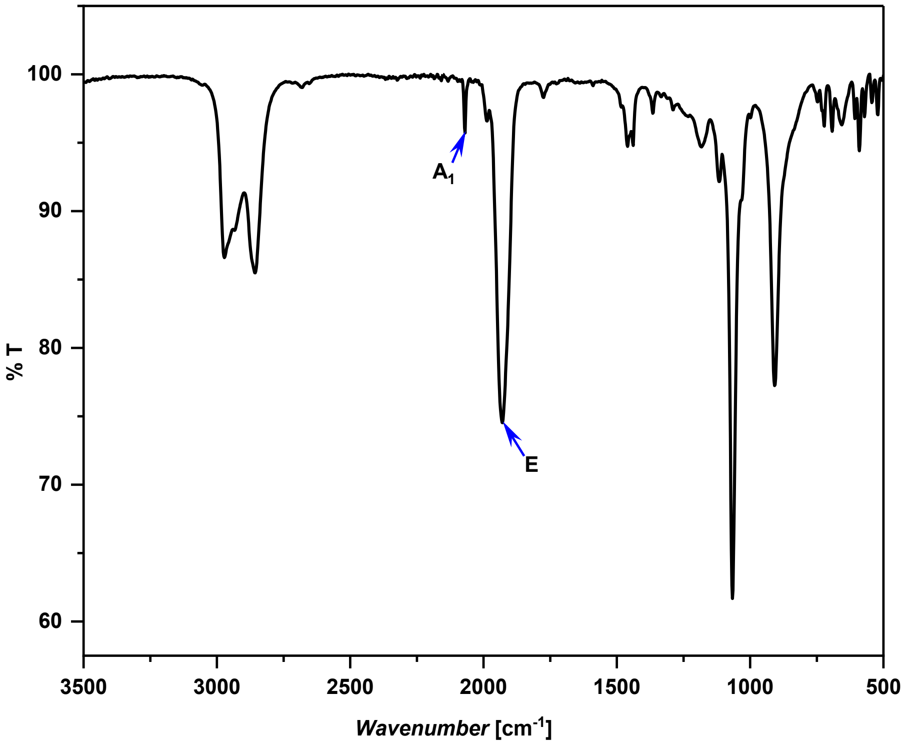

Figure S12. IR spectrum of **2** in THF.

2.3 $(CO)_5WCN_2PPh_3$: 3

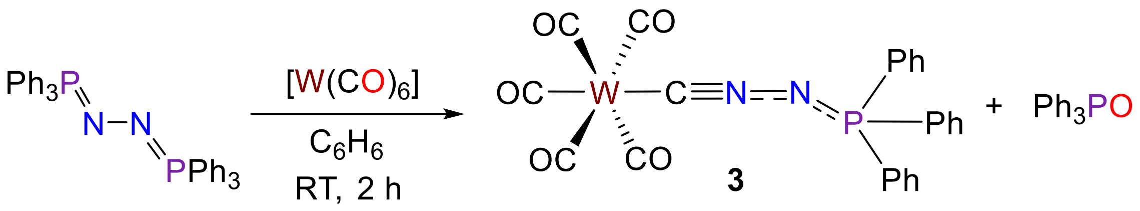

Inside the glovebox, a suspension of $[W(CO)_6]$ (41 mg, 0.12 mmol, 1.3 eq.) was stirred in a glass vial with red triphenylphosphineazine $[(Ph_3P)_2N_2]$ (50 mg, 0.09 mmol, 1.0 eq) dissolved in 5 mL of benzene. The mixture was stirred for 2 h, upon which the color of the reaction mixture changed from red to yellow. The crude $^{31}P\{^1H\}$ NMR spectrum revealed the formation of $[(CO)_5WCN_2PPh_3]$ and Ph_3PO in stoichiometric ratio (Figure S13). The reaction mixture was then filtered, and the solvent was removed *in vacuo* to afford a 1:1 mixture of **2** and Ph_3PO. Triphenylphosphine oxide can be removed through extraction by pentane (2×10 mL), followed by cooling the solution to –35 °C overnight to afford $[(CO)_5WCN_2PPh_3]$ (**3**)[3] as pale-yellow crystals in 67% yield (38 mg, 0.061 mmol). These crystals are suitable for sc-XRD.

Note: If running the reaction in stoichiometric ratio, a homogeneous solution was obtained, yet we preferred to run the reaction with a slight excess of tungsten hexacarbonyl due to the facile removal of this insoluble compound.

^{1}H NMR (C_6D_6, 400.1 MHz): δ = 7.47–7.41 (m, 6H, C*H*), 7.02–6.98 (m, 3H, C*H*), 6.96–6.91 (m, 6H, C*H*) ppm.

^{31}P{^{1}H} NMR (C_6D_6, 162.0 MHz): δ = 30.3 ($[W(CO)_5CN_2\textit{P}Ph_3]$), 27.1 ($Ph_3\textit{P}O$) ppm.

^{13}C{^{1}H} NMR (C_6D_6, 100.6 MHz): δ =198.3 (*C*O), 195.9 (*C*O), 133. 6 (d, $^4J_{CP}$ = 3 Hz, CH_{Ar}), 132.9 (d, $^3J_{CP}$ = 10 Hz, CH_{Ar}), 129.4 (d, $^2J_{CP}$ = 12 Hz, CH_{Ar}), 128.2 (d, $^1J_{CP}$ = 24 Hz, CH_{Ar}), 124.8 (d, $^1J_{W\text{–}C}$ = 97 Hz, WCN_2) ppm.[5]

m.p.: 115 °C.

IR in THF: $\tilde{\nu}_{CO}$ = 1937 cm^{-1} (vs, E), 2068 cm^{-1} (w, A_1).

CHN for $C_{24}H_{15}N_2O_5PW \cdot 0.2C_5H_{12}$ (isolated compound):

calcd. C 46.87, H 2.74, N 4.37;

found C 46.97, H 2.90, N 4.64.

HRMS (in THF, ESI^+) *m/z*: $[M+H]^+$ calcd. for $C_{24}H_{16}N_2O_5PW$ 627.0306; found 627.0320.

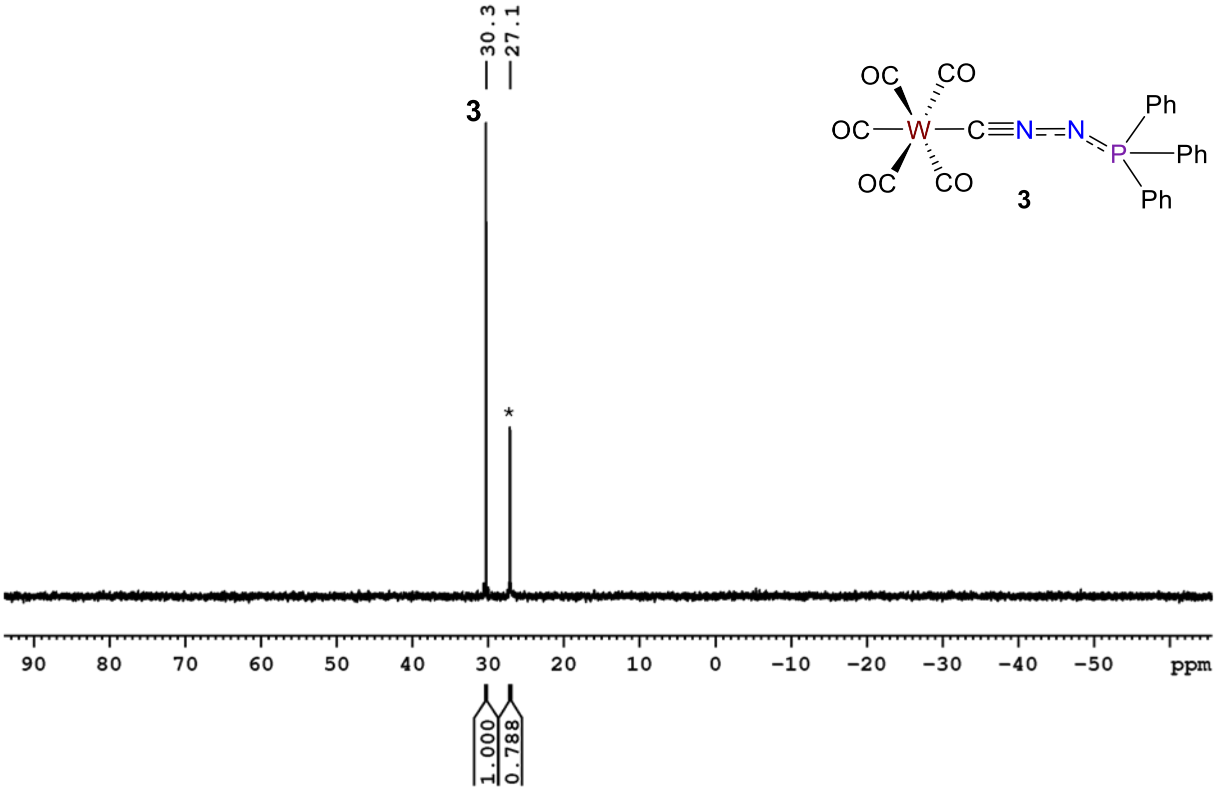

Figure S13. Crude $^{31}P\{^{1}H\}$ NMR spectrum obtained after stirring $[W(CO)_6]$ over a solution of $[(Ph_3P)_2N_2]$ for 2 h (C_6D_6, 162.0 MHz, D1 = 30 s, *Ph_3PO).

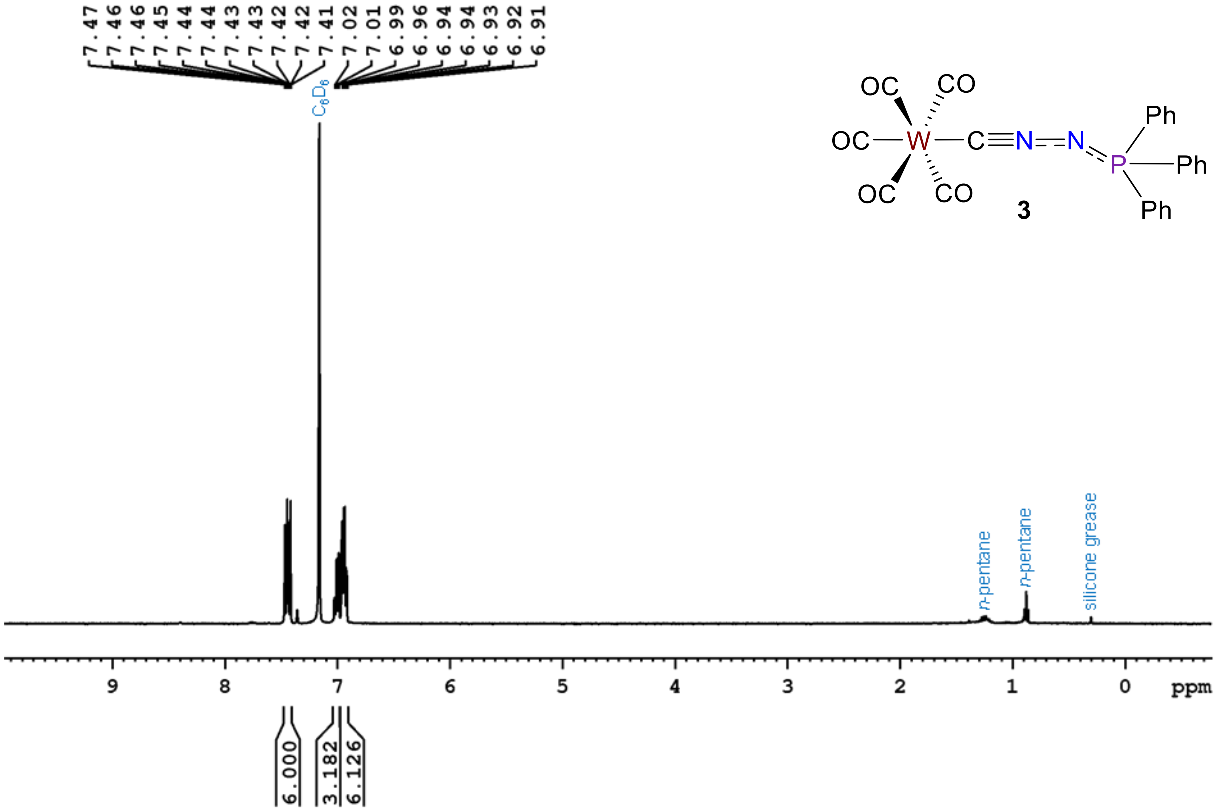

Figure S14. ^{1}H NMR spectrum of **3** (C_6D_6, 400.0 MHz).

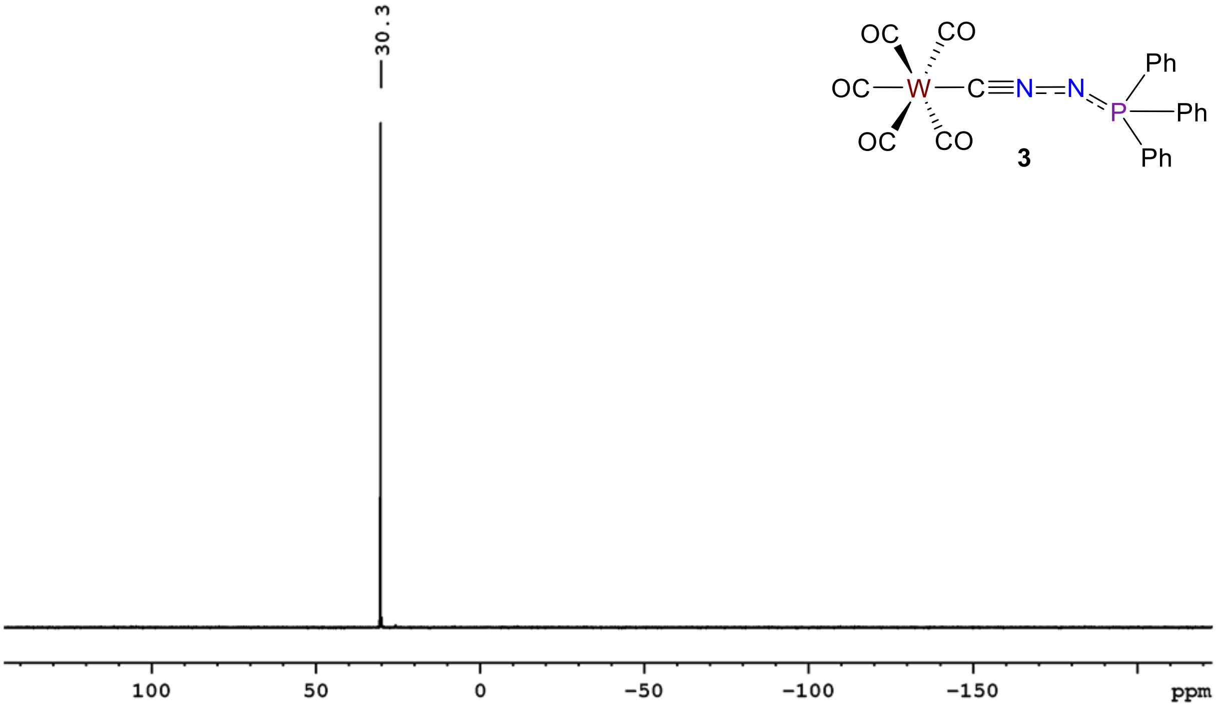

Figure S15. $^{31}P\{^1H\}$ NMR spectrum of **3** (C_6D_6, 162.0 MHz).

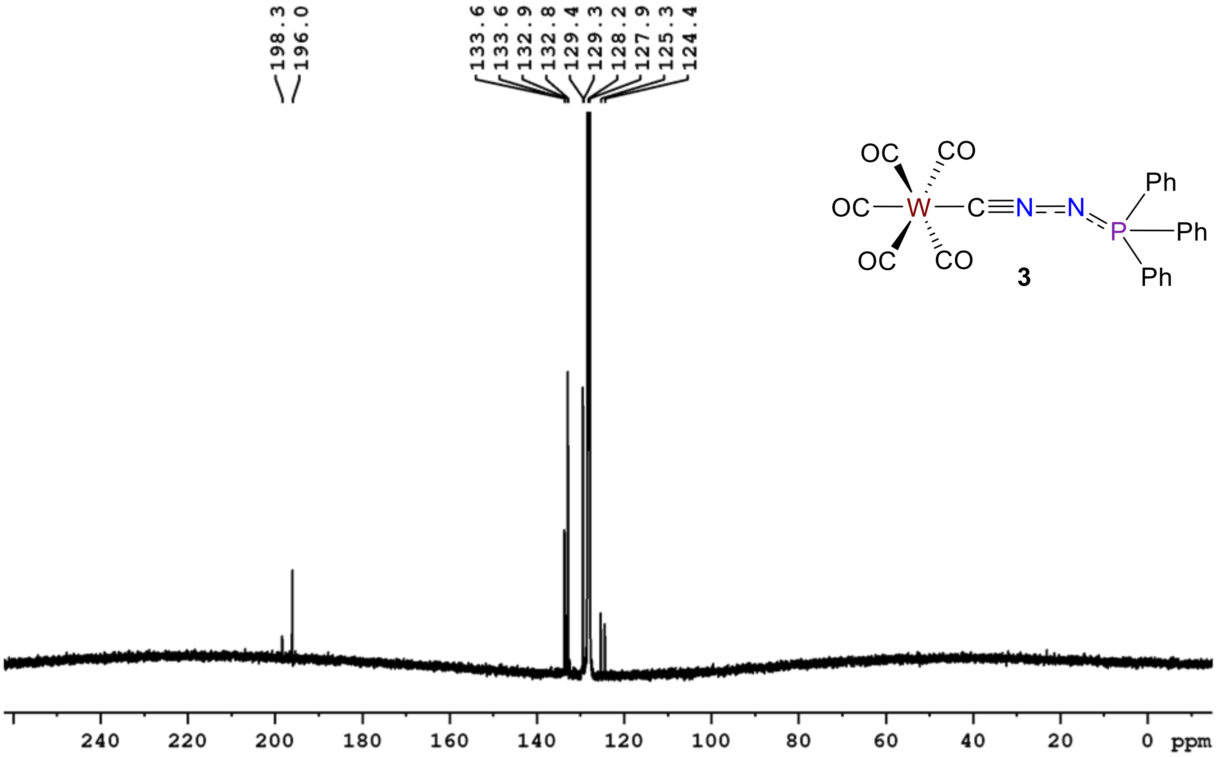

Figure S16. $^{13}C\{^1H\}$ NMR spectrum of **3** (C_6D_6, 100.6 MHz).

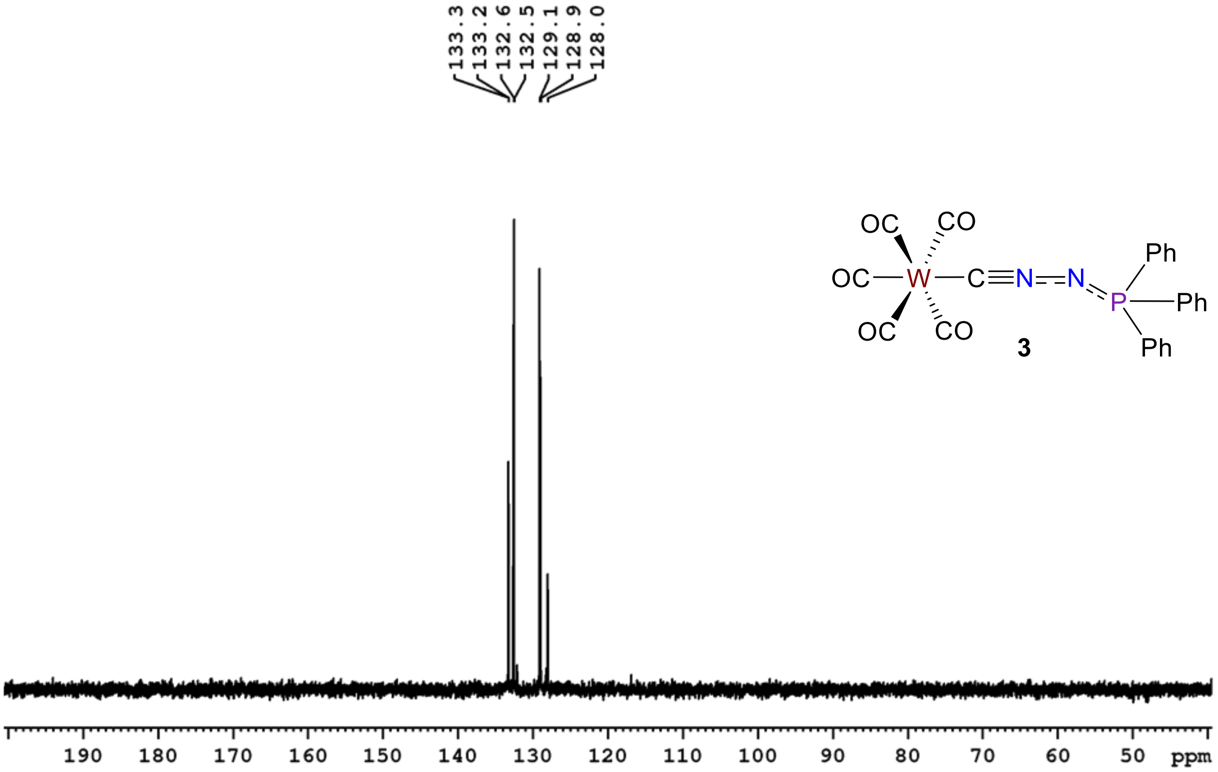

Figure S17. DEPT-135 NMR spectrum of **3** (C_6D_6, 100.6 MHz).

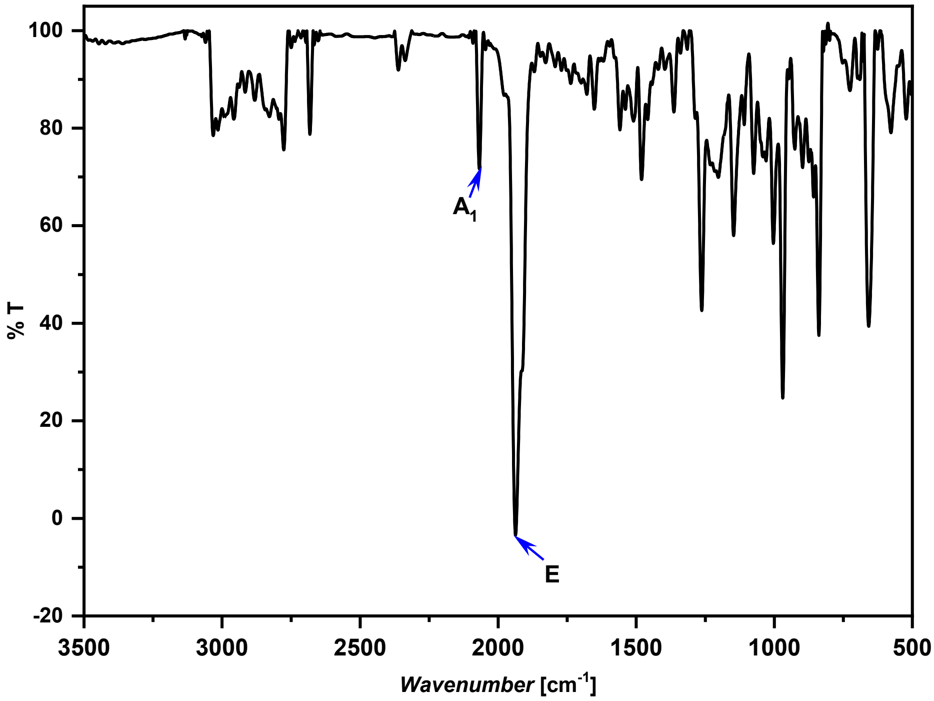

Figure S18. IR spectrum of **3** in THF.

.

2.4 $(CO)_4FeCN_2PPh_3$: 4

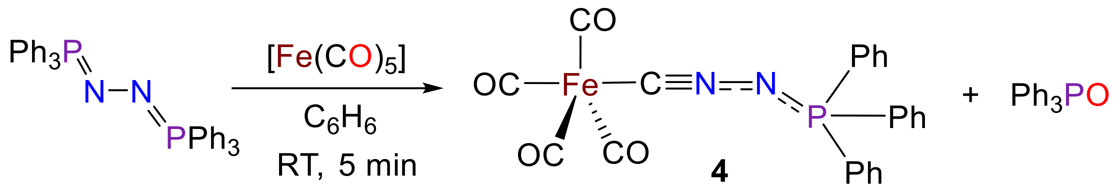

Inside the glovebox, $[Fe(CO)_5]$ (23 mg, 0.12 mmol, 1.3 eq, 16 µL) was mixed with red triphenylphosphineazine $[(Ph_3P)_2N_2]$ (50 mg, 0.09 mmol, 1.0 eq) dissolved in 5 mL of benzene in a glass vial. The color of the reaction mixture changed from red to yellow upon addition and was stirred for additional 30 min. The crude $^{31}P\{^1H\}$ NMR spectrum revealed the formation of $[(CO)_4FeCN_2PPh_3]$ and Ph_3PO in 1:1 ratio (Figure S19). The reaction mixture was then filtered, and the solvent was removed *in vacuo* to afford a 1:1 mixture of **2** and Ph_3PO (elemental analysis). Triphenylphosphine oxide can be removed by extraction with pentane (2×10 mL), followed by cooling the solution to –35 °C overnight to afford $[(CO)_4FeCN_2PPh_3]$ (**4**) as pale-yellow crystals in 73% yield (31 mg, 0.066 mmol). These crystals are suitable for sc-XRD.

^{1}H NMR (C_6D_6, 400.1 MHz): δ = 7.47–7.41 (m, 6H, C*H*), 7.03–6.98 (m, 3H, C*H*), 6.96–6.91 (m, 6H, C*H*) ppm.

^{31}P NMR (C_6D_6, 162.0 MHz): δ = 28.9 ppm.

^{13}C NMR (C_6D_6, 100.6 MHz): δ = 215.0 (CO), 214.9 (CO), 133. 5 (d, $^4J_{CP}$ = 2 Hz, CH_{Ar}), 132.9 (d, $^3J_{CP}$ = 10 Hz, CH_{Ar}), 129.4 (d, $^2J_{CP}$ = 12 Hz, CH_{Ar}), 128.1 (d, $^1J_{CP}$ = 23 Hz, CH_{Ar}), 125.0 (d, $^1J_{Fe–C}$ = 97 Hz, $FeCN_2$) ppm.[5]

m.p.: 104 °C.

IR (in THF): $\tilde{\nu}_{CO}$ = 1949 cm^{-1} (vs, E), 2056 cm^{-1} (s, A_1).

CHN for $C_{23}H_{15}N_2PO_4Fe \cdot C_{18}H_{15}PO \cdot 1C_6H_6$ (prior to pentane extraction):

calcd. C 68.29, H 4.39, N 3.39;

found C 67.85, H 4.90, N 3.59.

HRMS (in THF): (ESI^+) *m/z* $[M+H]^+$ calcd. for $C_{23}H_{16}FeN_2O_4P$ 471.0197; found 471.0429.

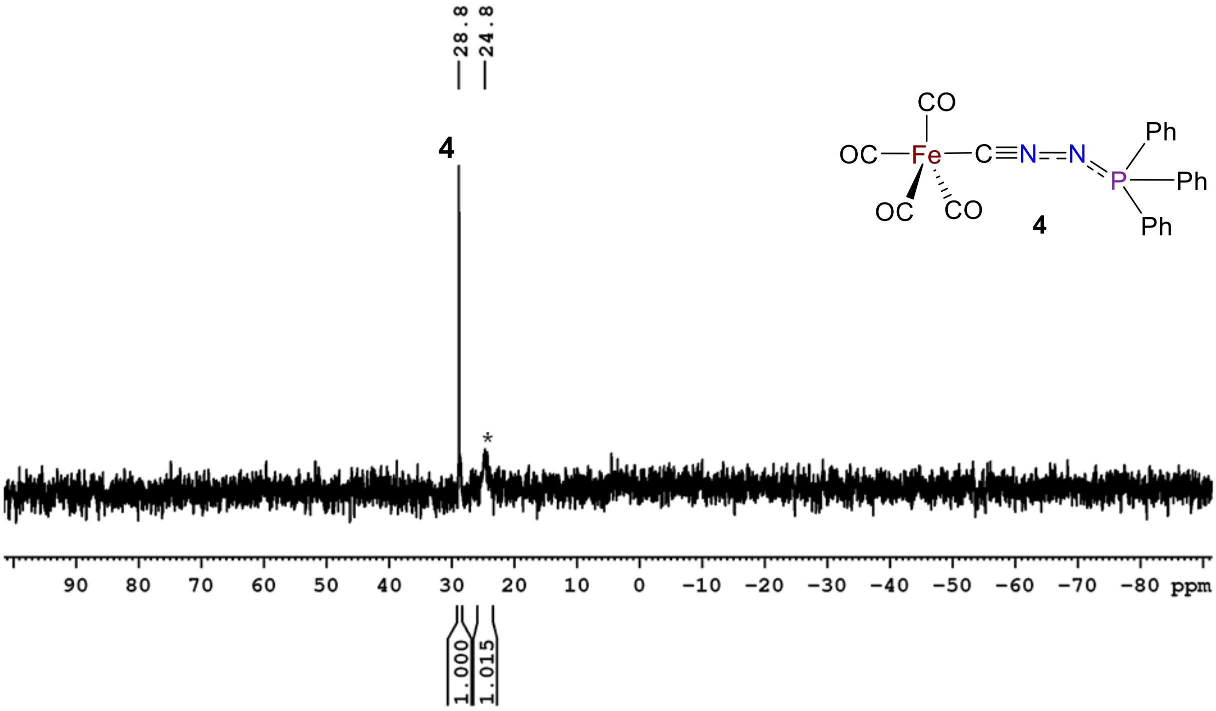

Figure S19. Crude $^{31}P\{^{1}H\}$ NMR spectrum obtained after stirring $[Fe(CO)_5]$ over a solution of $[(Ph_3P)_2N_2]$ after 10 min (C_6D_6, 162.0 MHz, D1 = 30 s, $^{*}Ph_3PO$).

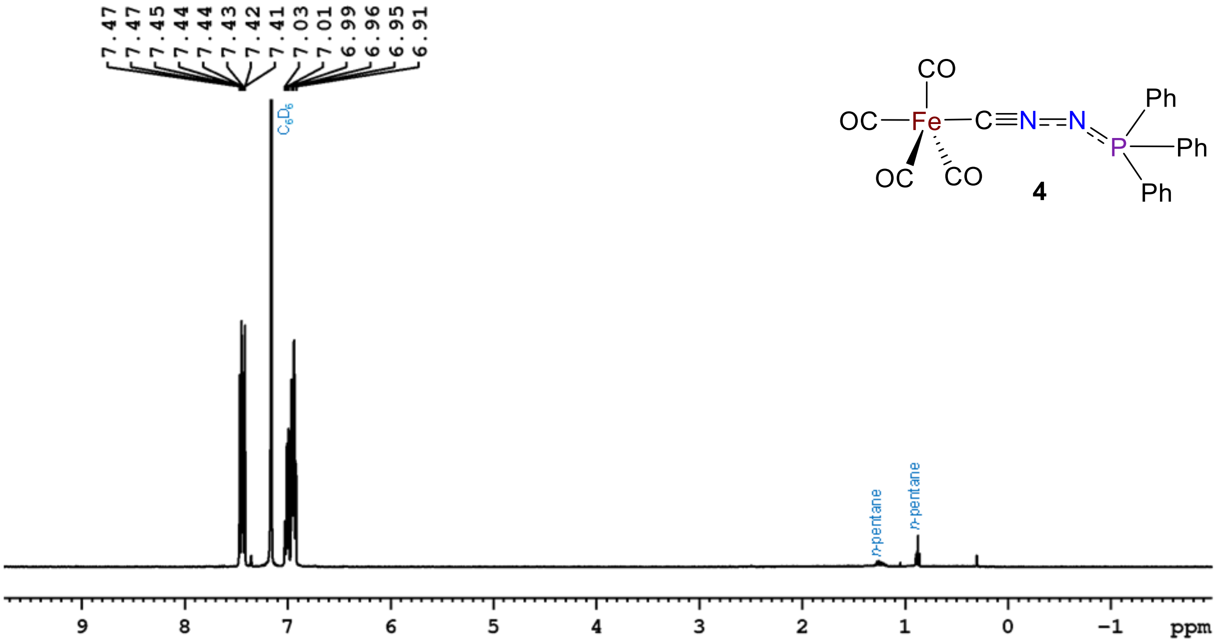

Figure S20. ^{1}H NMR spectrum of **4** (C_6D_6, 400.0 MHz).

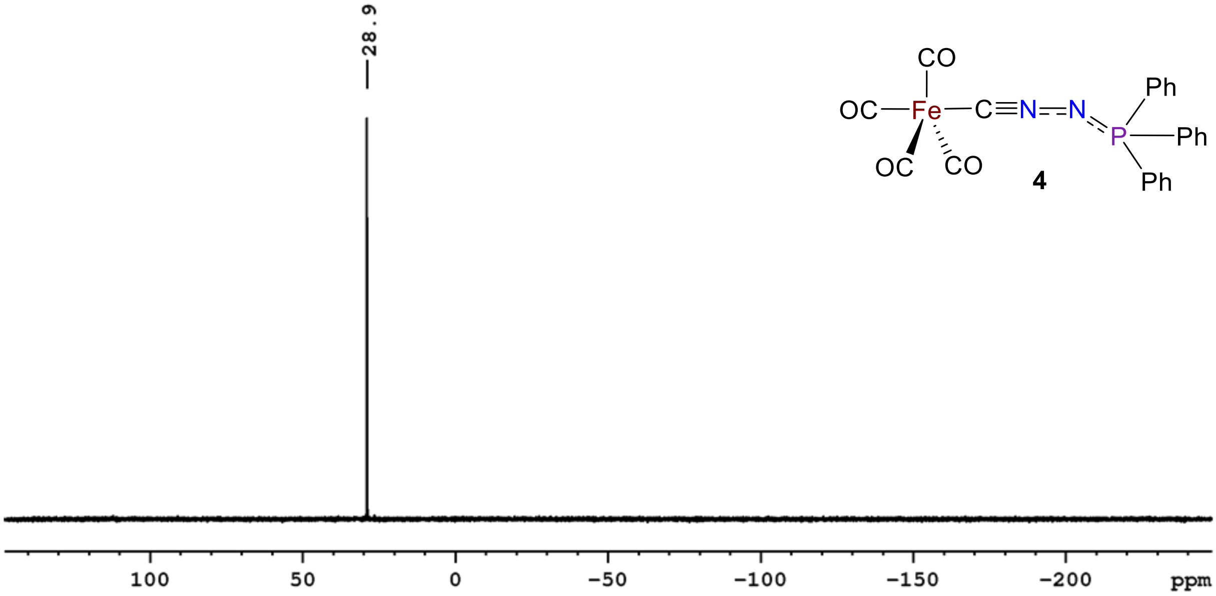

Figure S21. $^{31}P\{^{1}H\}$ NMR spectrum of **4** (C_6D_6, 162.0 MHz).

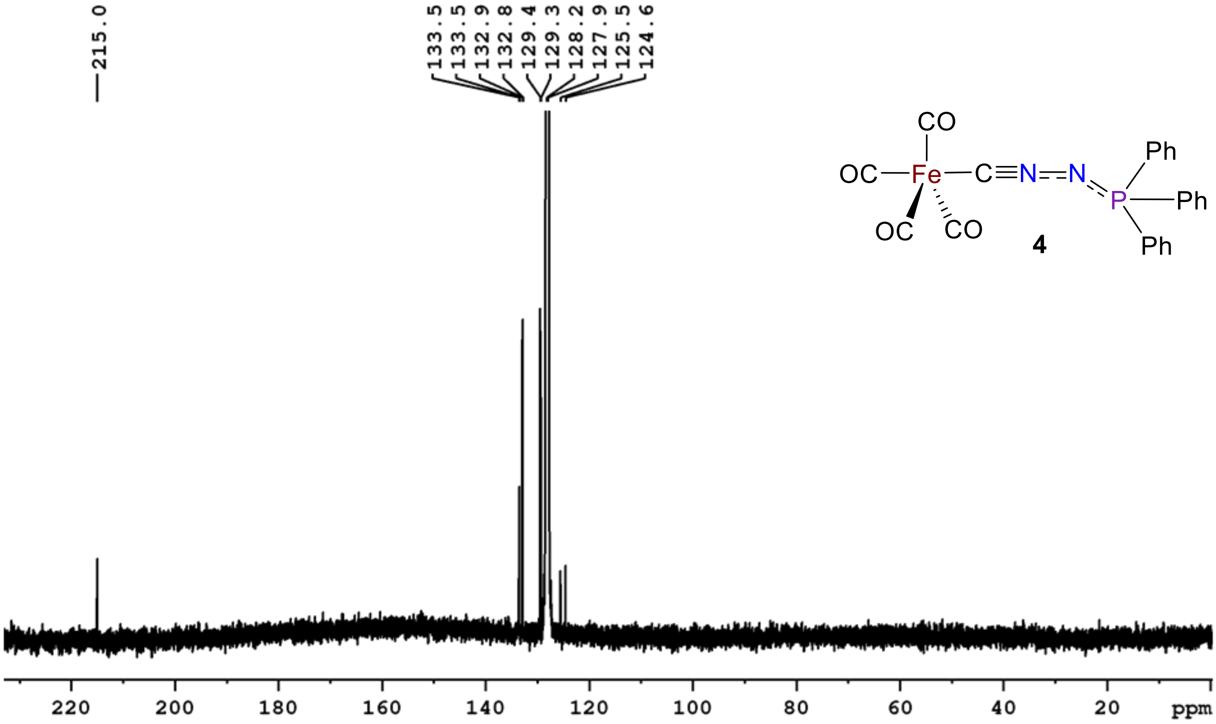

Figure S22. $^{13}C\{^{1}H\}$ NMR spectrum of **4** (C_6D_6, 100.6 MHz).

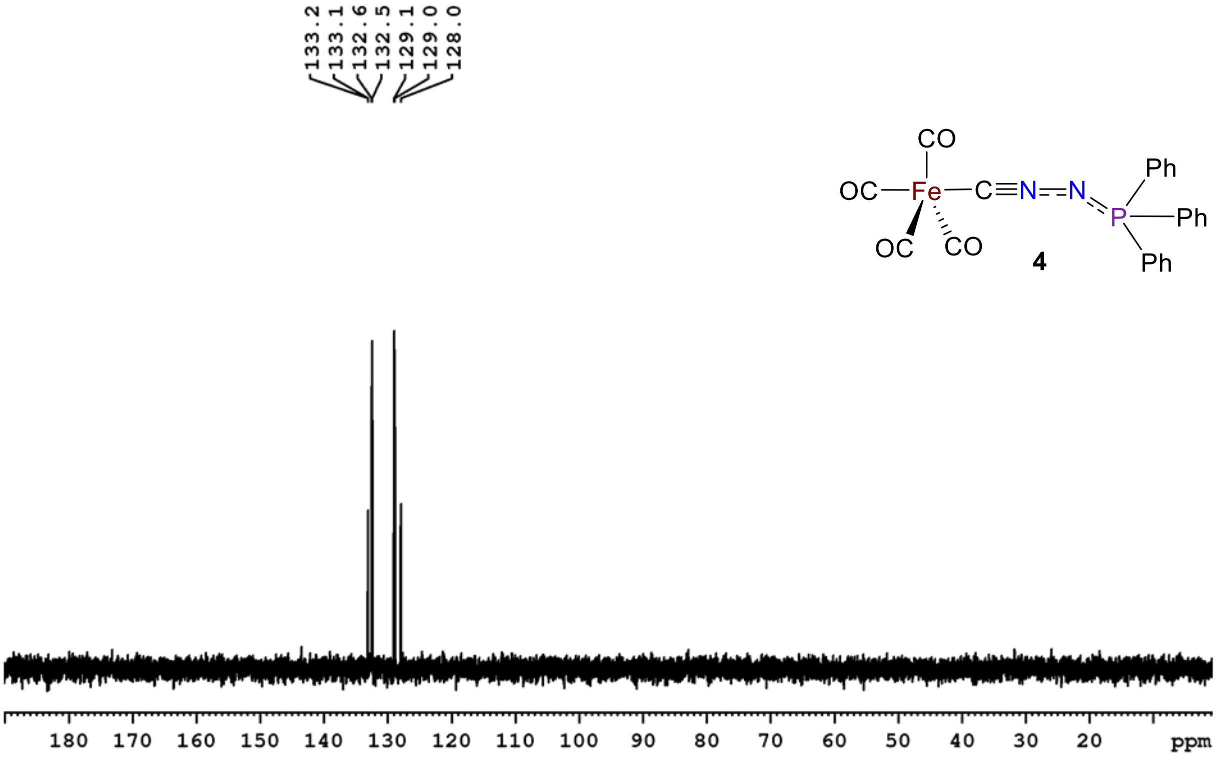

Figure S23. DEPT-135 NMR spectrum of **4** (C_6D_6, 100.6 MHz).

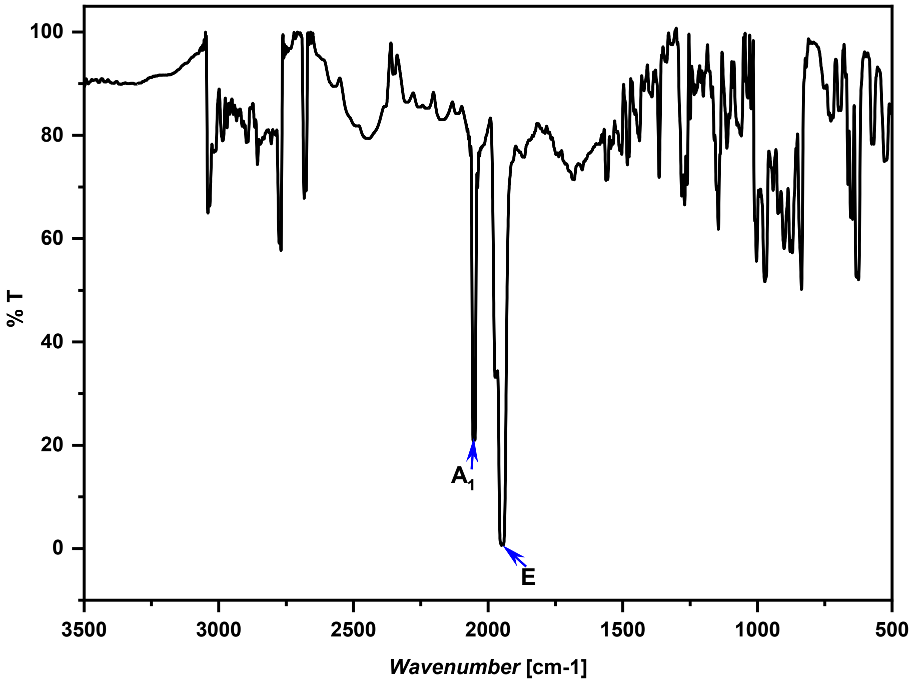

Figure S24. IR spectrum of **4** in THF.

3 Kinetic Studies

Inside the glovebox, suspensions of [M(CO)$_6$] (M = Cr, Mo, and W, 0.07 mmol, 1.3 eq) were stirred in glass vials with red triphenylphosphineazine [(Ph$_3$P)$_2$N$_2$] (30 mg, 0.05 mmol, 1.0 eq) dissolved in 3 mL of benzene. The reaction progress was monitored by ^{31}P{^{1}H} NMR spectroscopy in C$_6$H$_6$ (by taking out 70 µl aliquot each time). Reaction kinetics were determined by quantitative ^{31}P{^{1}H} NMR spectroscopy (D1 = 30 s). In sight of the quantitative nature of the reaction, no reference standards were used, and the concentrations were determined based on the relative integrated signal areas.

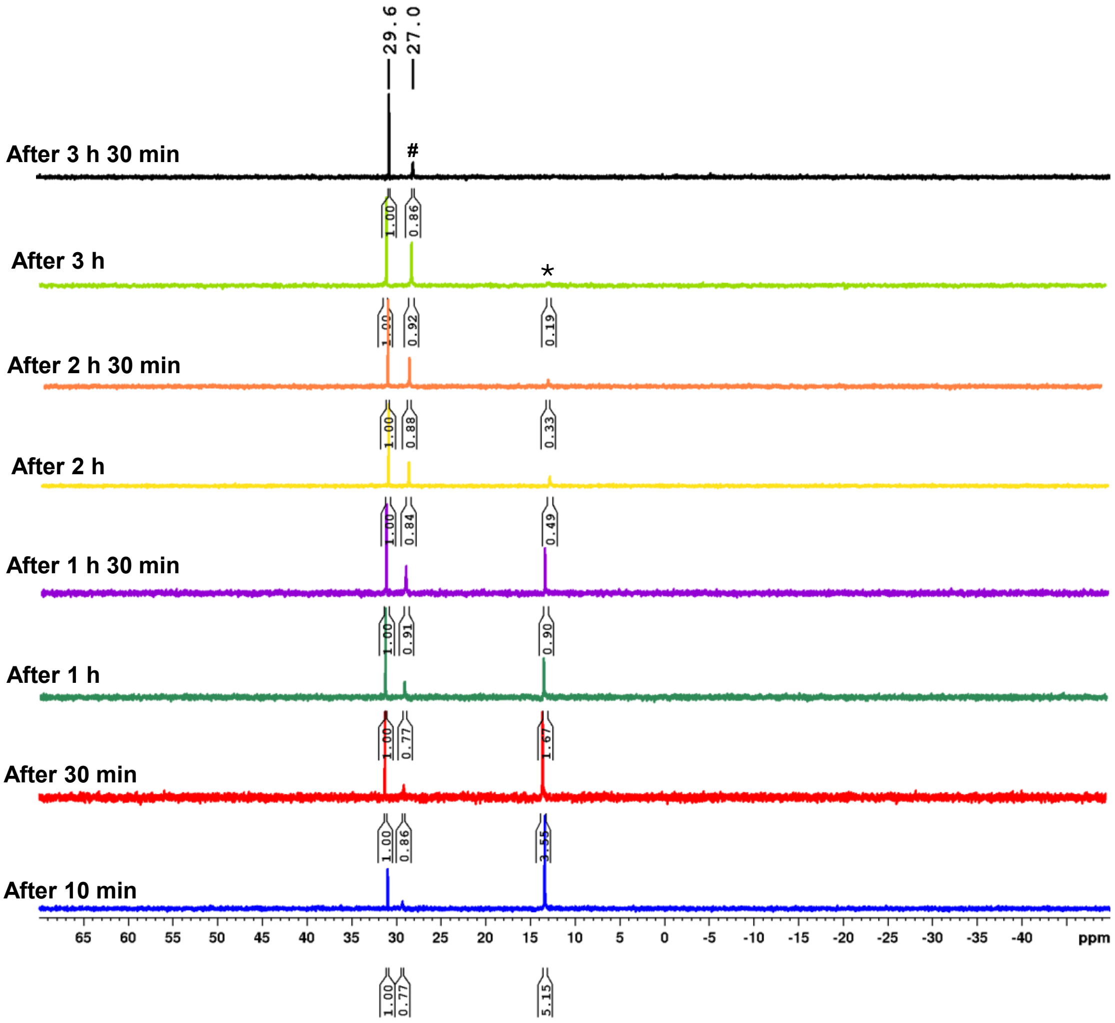

Figure S25. Stacked ^{31}P{^{1}H} NMR spectrum obtained upon stirring [Cr(CO)$_6$] in a solution of [(Ph$_3$P)$_2$N$_2$] in C$_6$H$_6$ over time (DMSO-d_6 capillary, 162.0 MHz, D1 = 30 s, *(Ph$_3$P)$_2$N$_2$ #Ph$_3$PO).

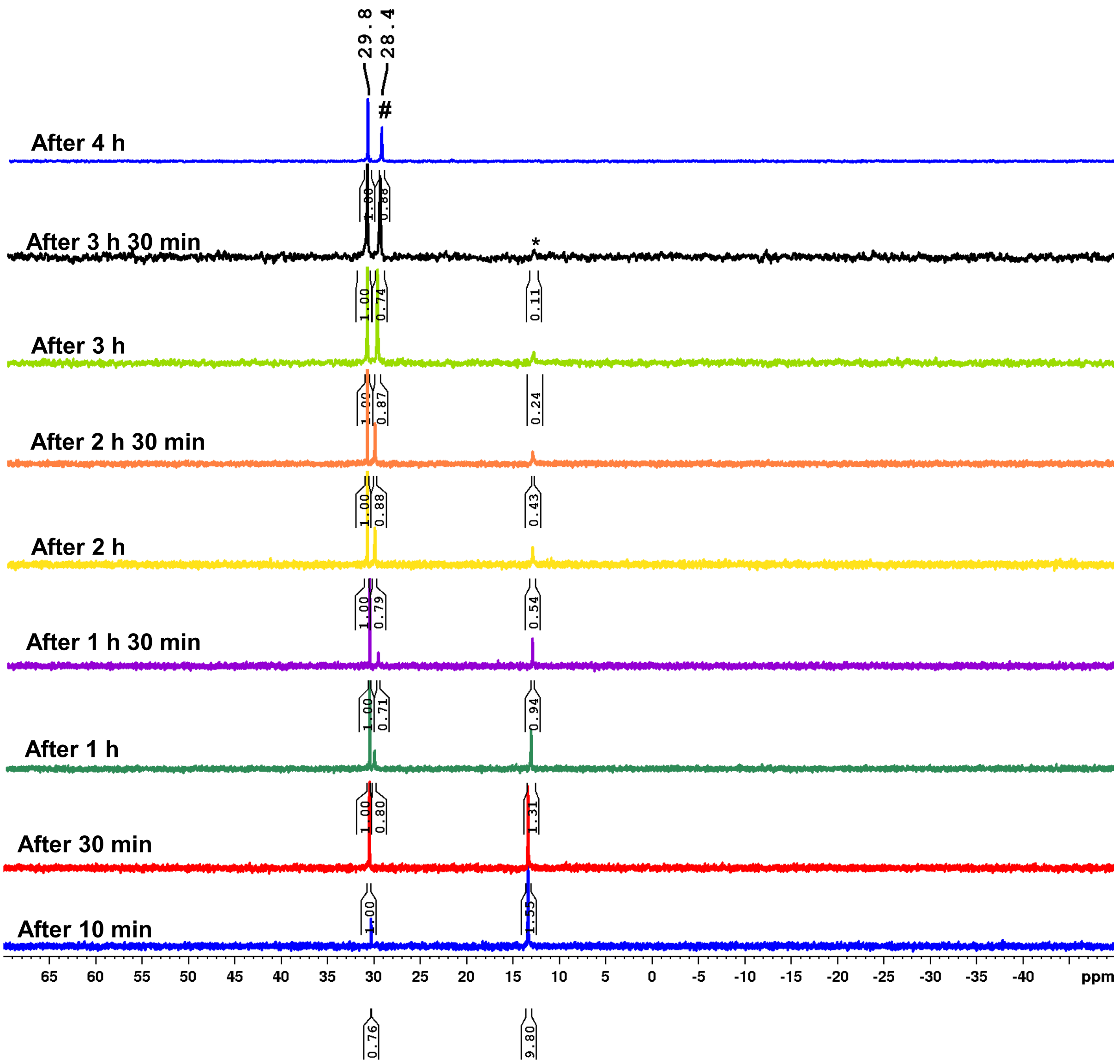

Figure S26. Stacked $^{31}P\{^1H\}$ NMR spectrum obtained upon stirring [Mo(CO)$_6$] in the solution of [(Ph$_3$P)$_2$N$_2$] over time (C_6H_6, DMSO-d_6 capillary, 162.0 MHz, D1 = 30 s, *(Ph$_3$P)$_2$N$_2$, #Ph$_3$PO).

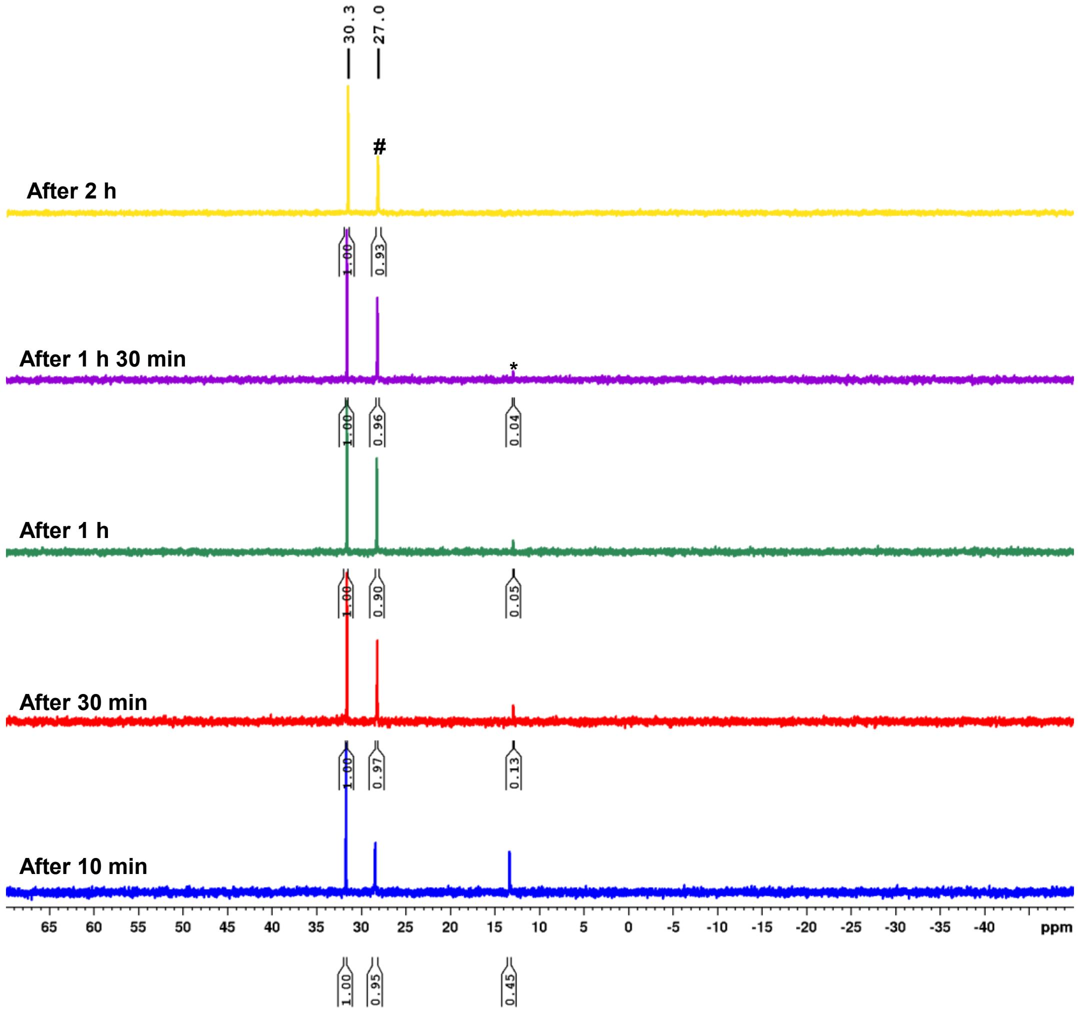

Figure S27. Stacked $^{31}P\{^1H\}$ NMR spectrum obtained upon stirring [$W(CO)_6$] in a solution of [$(Ph_3P)_2N_2$] over time (C_6H_6, DMSO-d_6 capillary, 162.0 MHz, D1 = 30 s, *$(Ph_3P)_2N_2$ #Ph_3PO).

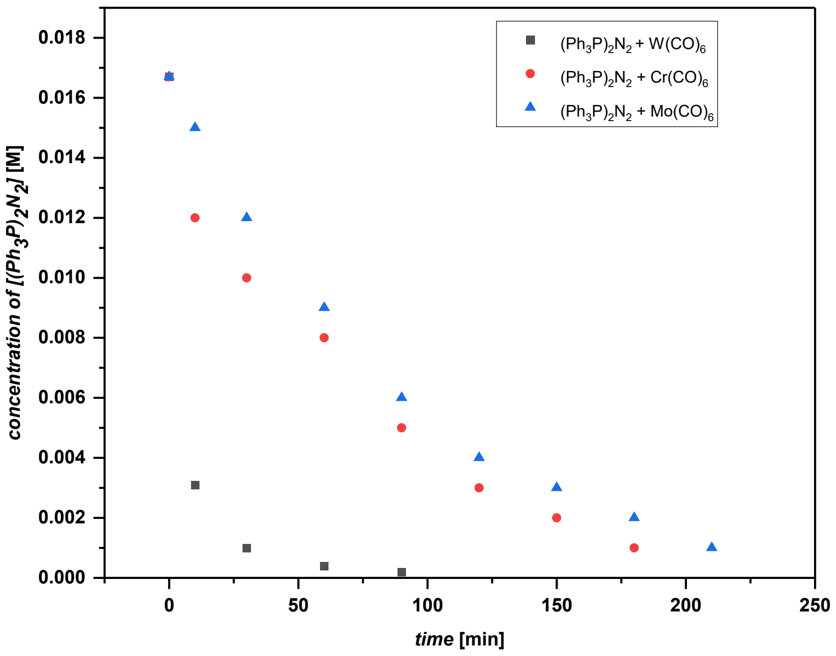

Figure S28. Conversion of [$(Ph_3P)_2N_2$] over time.

4 Experimental and Computed IR Data of 1–4

Table S1. Summary and assignment of experimental IR-stretching bands and comparison with DFT-computed stretching vibrations as obtained at the r^{2}SCAN-3c (ORCA v6.1.0) level of theory. Note that the triphenylphosphine-substituent effectively lowers the symmetry from idealized C_{4v} (complexes **1**–**3**; expected: 2 A_1 + E) and C_{3v} (complex **4**; expected: 2 A_1 + E), respectively. Further note that C_{4v} symmetric $M(CO)_5X$ complexes may show also a B_1 stretching mode in their infrared spectra.[6,7]

Complexes	Energy [cm^{-1}] and Intensity of Experimental IR Bands	Assignment	Energy [cm^{-1}] and Intensity of Computed IR Bands [cm^{-1}]	Assignment Computations
$Cr(CO)_5(CN_2PPh_3)$ (**1**)	n.a. (2141 Raman)[8]		2217 (weak)	C–N (A_1)
	2065 (weak)	A_1	2110 (moderate)	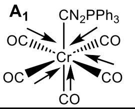
			2039 (moderate)	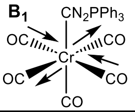
	1943 (very strong)	E	2012 (very strong)	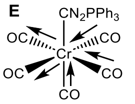
	1916 (strong)	E or B_1	2009 (very strong)	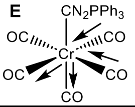
			1997 (very strong)	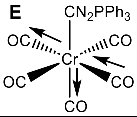
$Mo(CO)_5(CN_2PPh_3)$ (**2**)	n.a.		2207 (weak)	C–N (A_1)
	2069 (weak)	A_1	2112 (very strong)	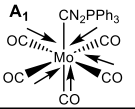
	1928 (very strong)	E	2038 (very strong)	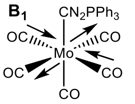
	1928 (very strong)	E	2008 (very strong)	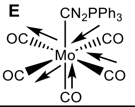
	ca. 1912 (shoulder)	E or B_1	2005 (very strong)	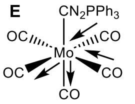
			1992 (very strong)	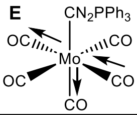
$W(CO)_5(CN_2PPh_3)$ (**3**)	n.a.		2208 (weak)	C–N (A_1)
	2068 (weak)	A_1	2111 (strong)	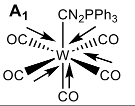

			2034 (strong)	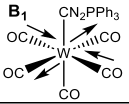
	1937 (very strong)	E	2003 (very strong)	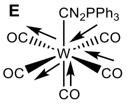
	1912 (strong)	E or B_1	2001 (very strong)	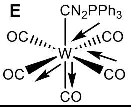
			1987 (very strong)	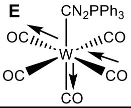
$Fe(CO)_4(CN_2PPh_3)$ (**4**)	n.a.		2248 (weak)	C–N (A_1)
	2056 (strong)	A_1	2099 (strong)	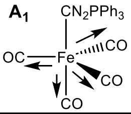
	1971 (strong)	E	2038 (strong)	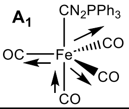
	1949 (very strong)	E	2013 (very strong)	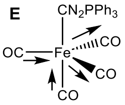
			1998 (very strong)	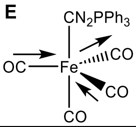
(CN_2PPh_3)[8]	2067	C–N		

5 Reaction of 1 and 3 with Iodosylbenzene

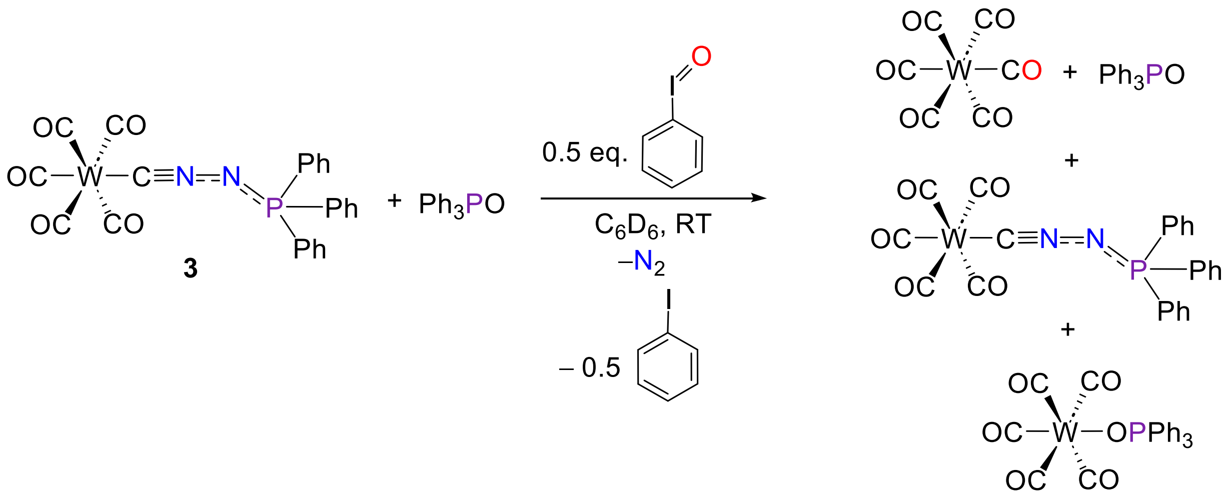

Inside a glovebox, a freshly prepared 1:1 mixture of [(CO)$_5$WCN$_2$PPh$_3$] **3** (21 mg, 33.5 µmol) and Ph$_3$PO (9 mg, 32.3 µmol; total mixture mass 30 mg) was dissolved in C_6D_6 (0.6 mL) in a J. Young NMR tube. To the resulting pale-yellow solution, iodosylbenzene, PhIO (4 mg, 16.8 µmol, 0.5 equiv.) was added as a suspension. The reaction mixture was then sonicated for 20 min. After sonication, the crude mixture was analysed by ^{31}P{^{1}H} NMR spectroscopy. Then, the mixture was transferred to a small Teflon-capped vial and stirred. During stirring, the evolution of N_2 gas was observed. After stirring overnight, the reaction mixture gradually changed from pale yellow to brown. The crude product mixture was analyzed by ^{31}P{^{1}H} NMR and IR spectroscopies. The ^{31}P{^{1}H} NMR spectrum of the crude reaction mixture indicated the formation of [W(OPPh$_3$)(CO)$_5$] along with unreacted starting material and Ph$_3$PO (Figures S29, S30). The formation of [W(OPPh$_3$)(CO)$_5$] was confirmed by irradiating a mixture of W(CO)$_6$ and Ph$_3$PO by a Xe lamp (Quantum Design, LSE140/160.25C, 150 W). The ^{31}P{^{1}H} NMR chemical shift of 43.7 ppm in *o*DFB is consistent with δ(^{31}P, THF) = 43.6 ppm reported in the literature (Figures S29 and S31).[9, 10] The IR spectroscopic analysis (Figure S32) revealed the formation of W(CO)$_6$.

Note: The equivalent reactivity was observed for **1** as well as in reaction with silver(I) oxide.

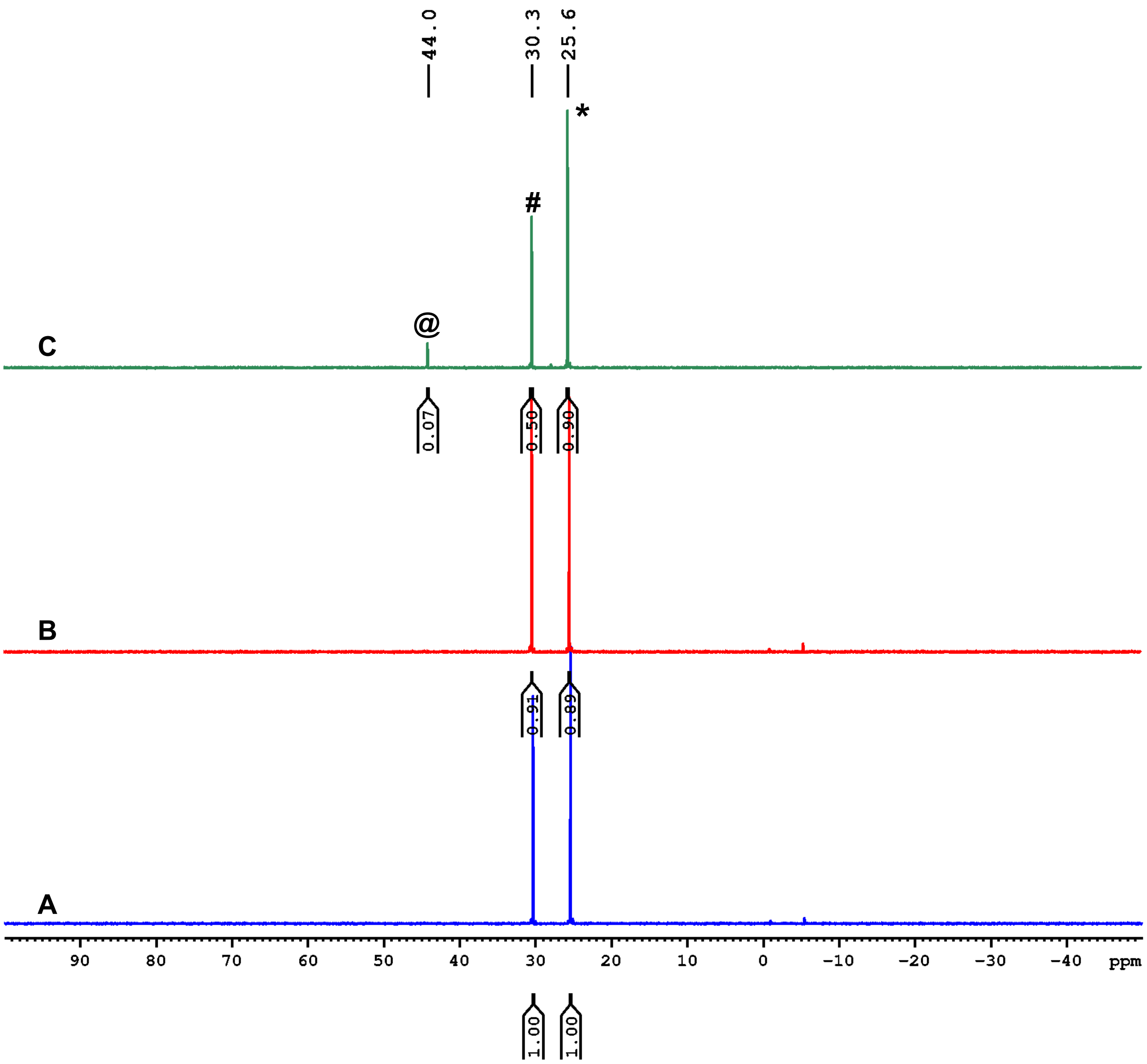

Figure S29. Stacked crude $^{31}P\{^1H\}$ NMR spectra: **A**. Before addition of iodosylbenzene (PhIO) in a 1:1 mixture of $[(CO)_5WCN_2PPh_3]$ **3** and Ph_3PO; **B**. After suspending PhIO and sonication for 20 min; **C**. After stirring the reaction mixture overnight (C_6D_6, 162.0 MHz, D1 = 30 s, $^{@}(CO)_5W(OPPh_3)$, $^{\#}[(CO)_5WCN_2PPh_3]$, $^{*}Ph_3PO$). Note that the reaction had been performed with crude complex **3**, which still contained 1 equivalent of Ph_3PO.

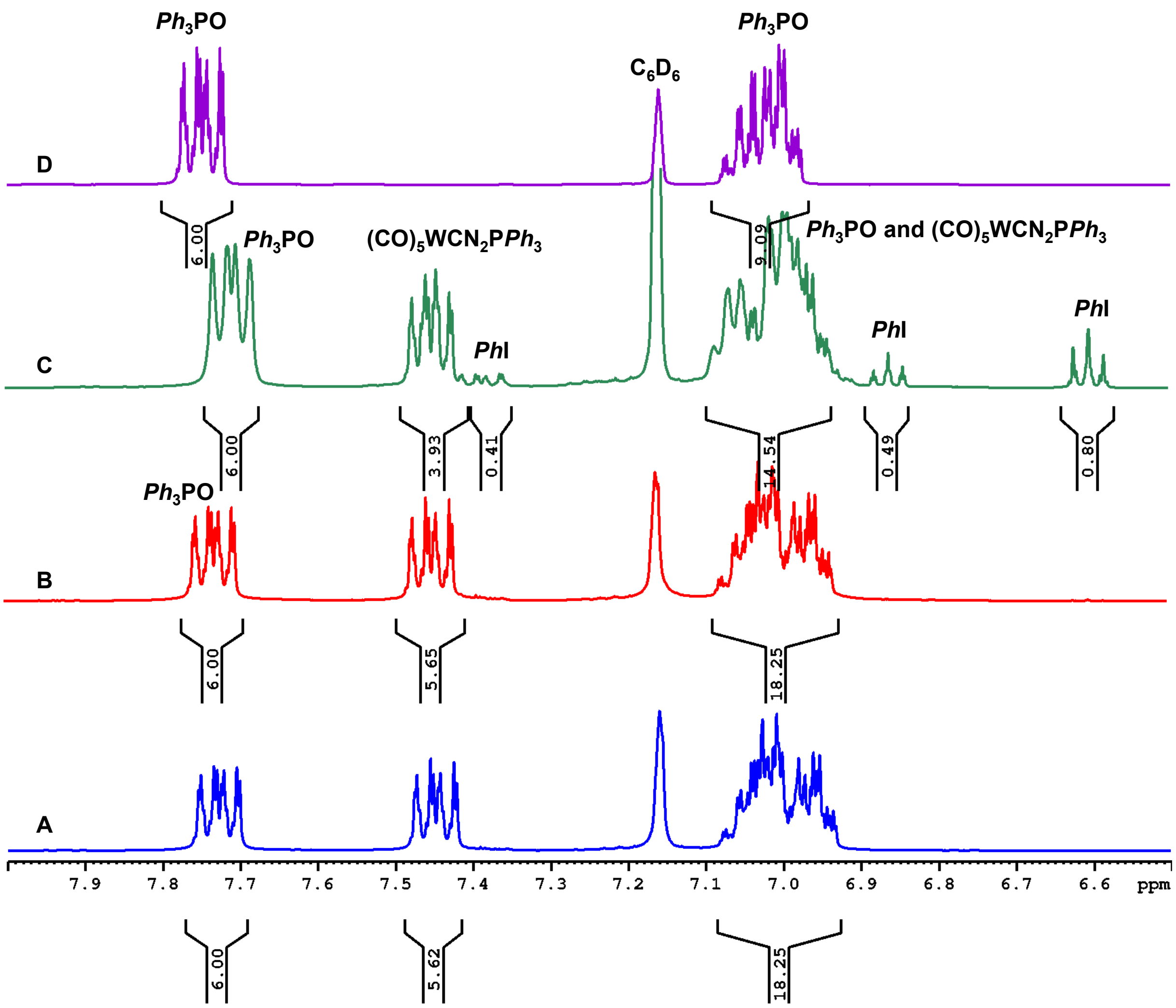

Figure S30. Stacked ^{1}H NMR spectra: **A**. Before addition of iodosylbenzene (PhIO) in a 1:1 mixture of [(CO)$_5$WCN$_2$PPh$_3$] **3** and Ph$_3$PO; **B**. After suspending PhIO and sonication for 20 min; **C**. After stirring the reaction mixture overnight; **D**. reference spectrum of Ph$_3$PO (C_6D_6, 400.0 MHz).

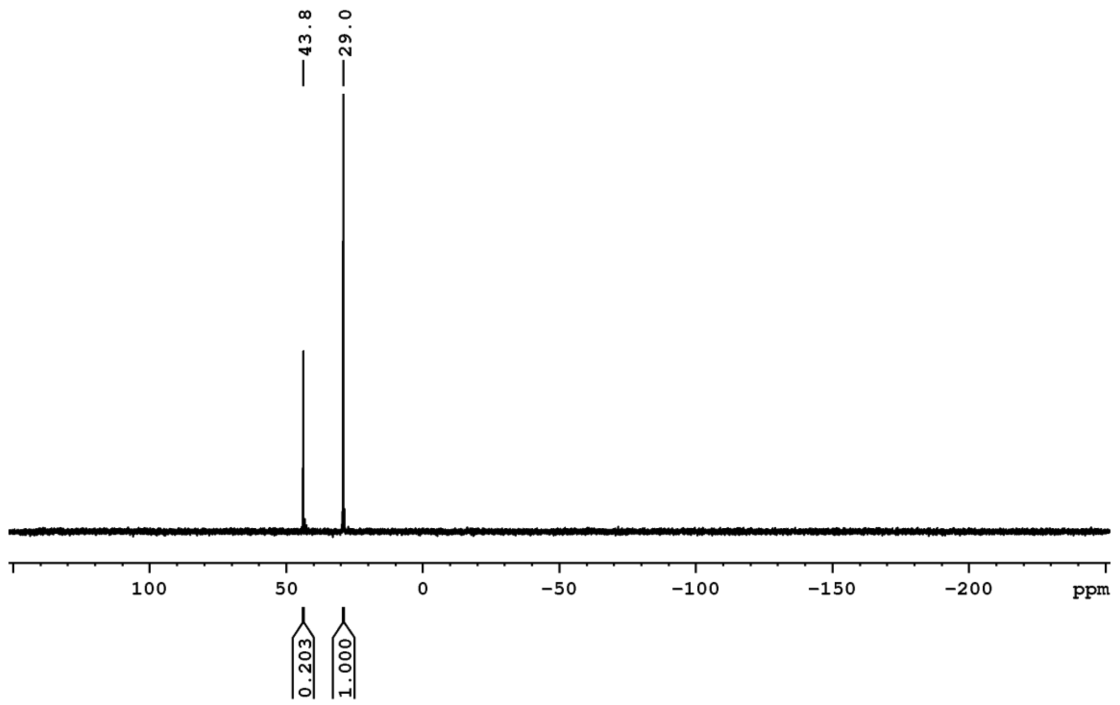

Figure S31. Crude $^{31}P\{^{1}H\}$ NMR spectrum obtained after irradiation of $W(CO)_6$ with Ph_3PO for 20 min ($CDCl_3$, 121.5 MHz, D1 = 30s).[10]

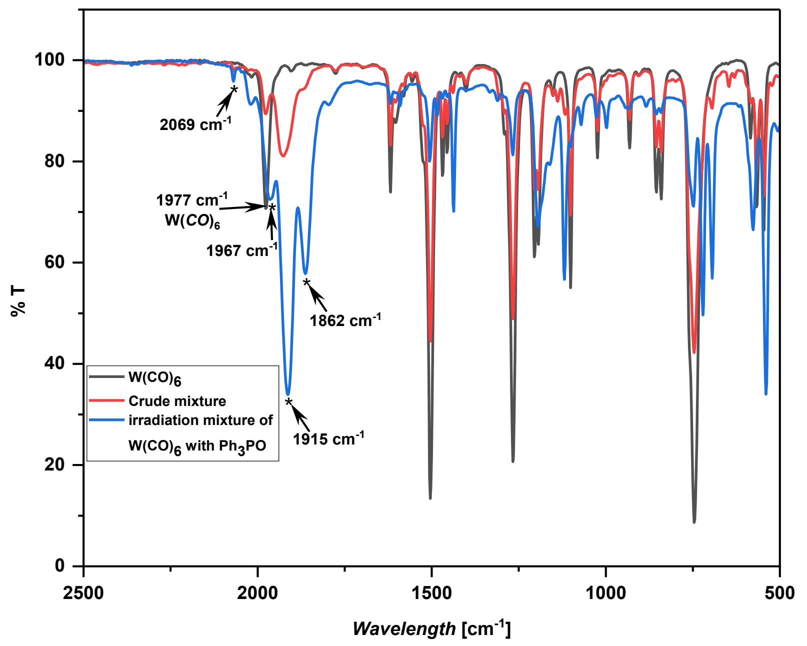

Figure S32. Stacked IR spectra of the crude oxidation reaction (red), $W(CO)_6$ (black), and the yellow mixture obtained after irradiating $W(CO)_6$ with Ph_3PO under a Xe lamp for 20 min (blue) in *o*DFB. *assigned as $\tilde{\nu}(CO)$ of $(CO)_5W(OPPh_3)$ from the literature reported $\tilde{\nu}$ (CO, THF) 2069.5 w, 1974.1 w, 1924.9 s, 1875.7 m.[10]

6 Reaction of 1 with [Me_4NF]

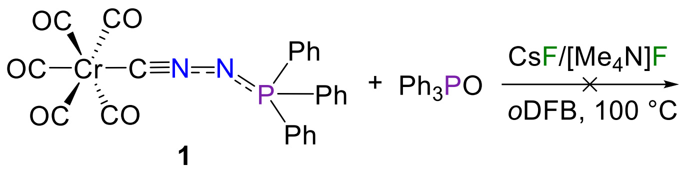

Inside the glovebox, a freshly prepared mixture of [$(CO)_5CrCN_2PPh_3$] **1** and Ph_3PO (1:0.4 molar ratio; 5 mg, 10 μmol) was dissolved in *ortho*-difluorobenzene (*o*DFB) in a J. Young NMR tube. To this solution, CsF (1 mg, 8 μmol, 1.0 equiv) was suspended and the reaction mixture was sonicated at room temperature for 20 min. Due to the low solubility of CsF in *o*DFB, 1 equiv. of [Me_4N]F (1 mg, 8 μmol, 1.0 equiv.) was added. The resulting reaction mixture was analyzed by $^{31}P\{^1H\}$ NMR spectroscopy, which showed only the presence of starting material after 1 h of sonication (Figure S33). The mixture was then refluxed at 100 °C for 2 h, after which the $^{31}P\{^1H\}$ NMR spectroscopic analysis indicated the presence of triphenylphosphine oxide (Ph_3PO) along with a peak at δ = 27 ppm, which is assigned as free *N*-isocyaniminotriphenylphosphorane according to the literature[11] [$\delta(^{31}P, CDCl_3)$ = 29 ppm], along with trace amount of starting material. The CN/NC-isomerized product, free *N*-cyaniminophosphorane, is expected to appear at 23.1 ppm in the $^{31}P\{^1H\}$ NMR spectrum in $CDCl_3$.[12]

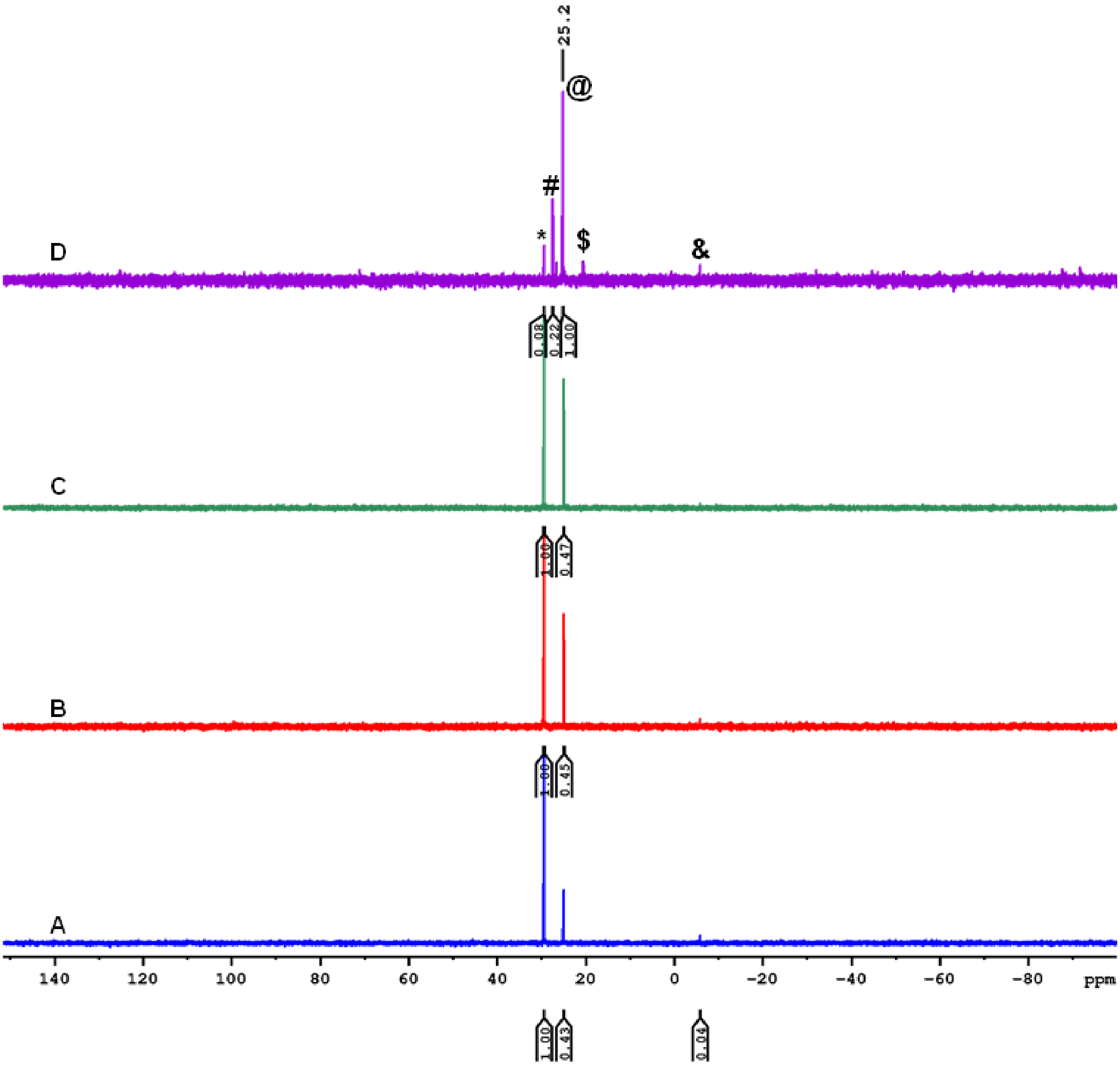

Figure S33. Crude $^{31}P\{^1H\}$ NMR spectrum obtained after addition of CsF/Me_4NF in [$(CO)_5CrCN_2PPh_3$] **1** (*o*DFB with DMSO-d_6 capillary, 121.5 MHz, D1 = 30s). **A.** Before the addition of CsF/Me_4NF; B. After the addition of CsF/Me_4NF, sonicated for 1 h; **C.** After additional sonication for 2 h; **D.** After refluxing the reaction mixture for 2 h; *$(CO)_5CrCN_2PPh_3$ **1**, #assigned as free ligand, *N*-isocyaniminotriphenyl-phosphorane[11] $\delta(^{31}P, CDCl_3)$: 29 ppm, @Ph_3PO, $unidentified phosphorus species, &PPh_3.

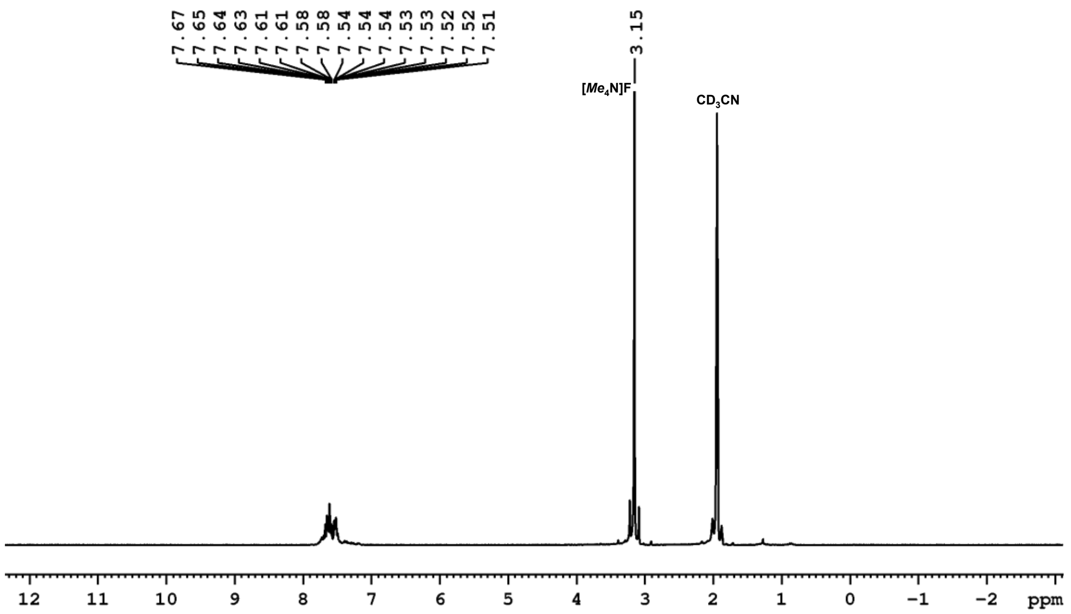

Figure S34. Crude ^{1}H NMR spectrum obtained after addition of CsF/Me_4NF in $[(CO)_5CrCN_2PPh_3]$ **1** (CD_3CN, 300.1 MHz).

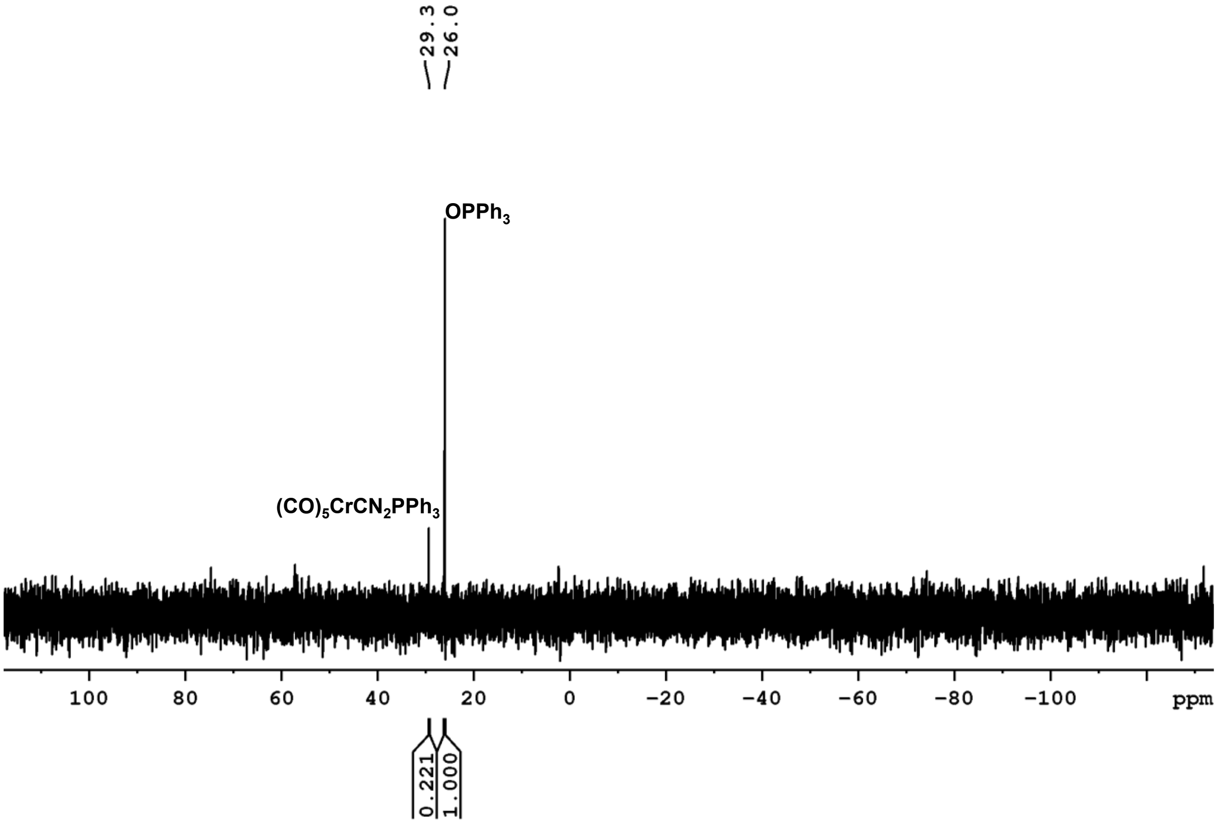

Figure S35. Crude ^{31}P{^{1}H} NMR spectrum obtained after refluxing of CsF/Me_4NF with $[(CO)_5CrCN_2PPh_3]$ (CD_3CN, 121.5 MHz, D1 30s).

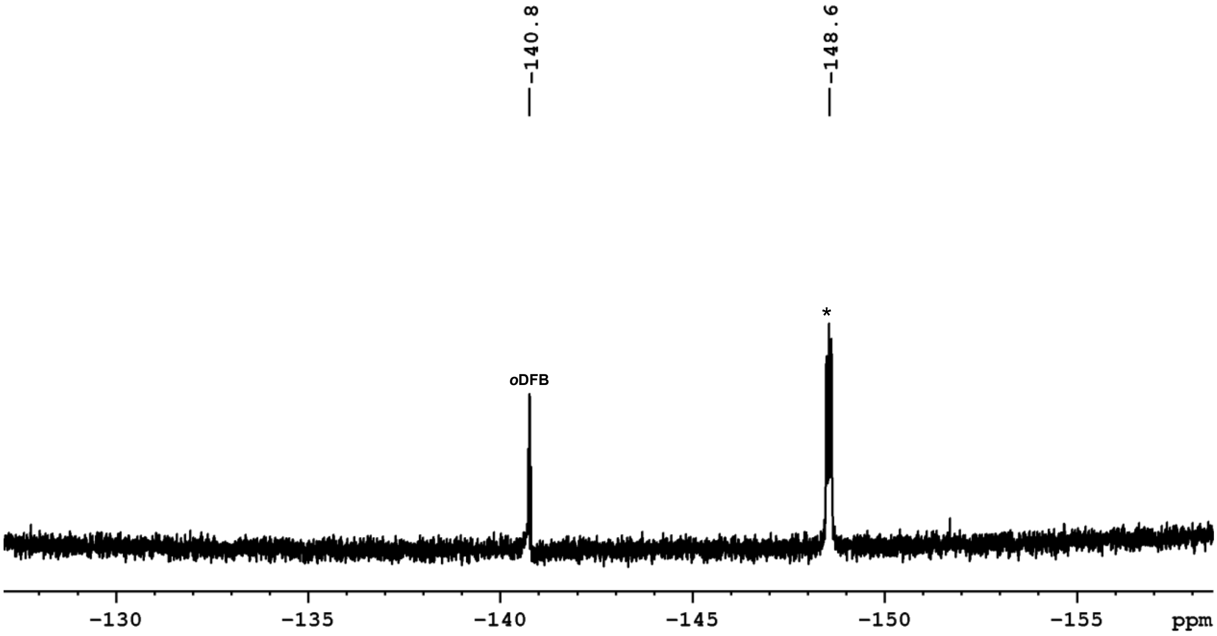

Figure S36. Crude ^{19}F NMR spectrum obtained after refluxing of CsF/Me_4NF with [$(CO)_5CrCN_2PPh_3$] (CD_3CN, 282.4 MHz, D1 30s, *assigned as DF_2^-: Me_4NF deprotonates CD_3CN over the course of several hours, thereby generating DF_2^-)[13].

7 Attempts Toward bis-*aza*-Wittig Reaction

Reaction of [$(Ph_3P)_2N_2$] with [$W(CO)_6$] in *o*DFB or THF-d_8

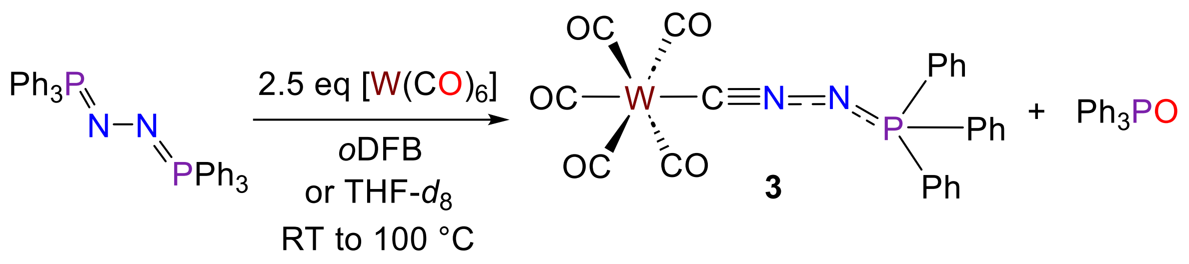

Inside the glovebox, red triphenylphosphineazine [$(Ph_3P)_2N_2$] (30 mg, 0.05 mmol, 1.0 equiv.), was dissolved in 0.6 mL of either *o*DFB or THF-d_8 in separate *J*. Young NMR tubes. [$W(CO)_6$] (48 mg, 0.14 mmol, 2.5 equiv.) was added. The suspension was sonicated for 10 min, during which the color changed from red to yellow for both solvents. Analysis of the crude $^{31}P\{^1H\}$ NMR spectrum revealed the formation of [$(CO)_5WCN_2PPh_3$] (**3**) and Ph_3PO in a 1:1 ratio.

Subsequently, the reaction mixture was sonicated at 60 °C for 2 h, affording a homogeneous yellow solution. The crude $^{31}P\{^1H\}$ NMR spectrum revealed however no change in the ratio of [$(CO)_5WCN_2PPh_3$] (**3**) to Ph_3PO (Figures S37, S38). Further heating to 80 °C for 2 h likewise resulted in no change in product distribution according to $^{31}P\{^1H\}$ and ^1H NMR (Figure S39) spectroscopies. Refluxing the crude reaction mixture at 100 °C for an additional 2.5 h also failed to substantially alter the ratio of [$(CO)_5WCN_2PPh_3$] (**3**) and Ph_3PO according to $^{31}P\{^1H\}$ NMR spectroscopy; in the case of THF solvent, a minor amount (<10%) of an unidentified phosphorus-species formed (Figure S38).

Note: The first aza-Wittig reaction proceeds more rapidly in THF than in *o*DFB (and benzene). In THF-d_8, sonication at room temperature for 5 min was sufficient to induce a color change from red to yellow, hence indicating quantitative conversion within 5 min, whereas 10 min are required for *o*DFB.

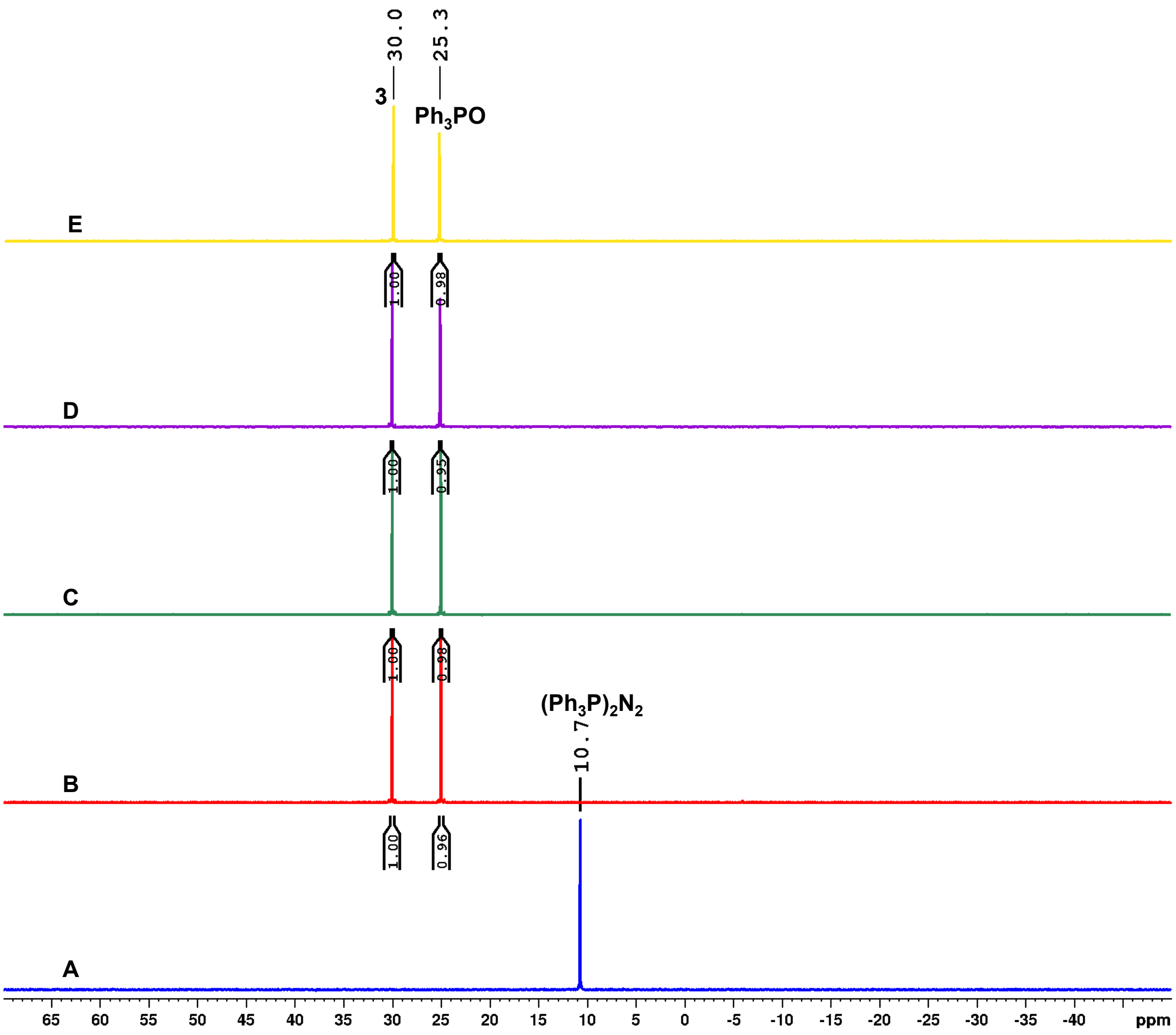

Figure S37. Stacked crude $^{31}P\{^{1}H\}$ NMR spectra (*o*DFB with DMSO-*d*$_6$, 162.0 MHz, D1 = 30 s): **A**. [$(Ph_3P)_2N_2$] in *o*DFB; **B**. After sonicating [$W(CO)_6$], suspension for 10 min at RT; **C**. After sonication for 2 h at 60 °C; **D**. After heating to 80 °C for 2 h; **E**. After refluxing the reaction mixture at 100 °C for 2.5 h.

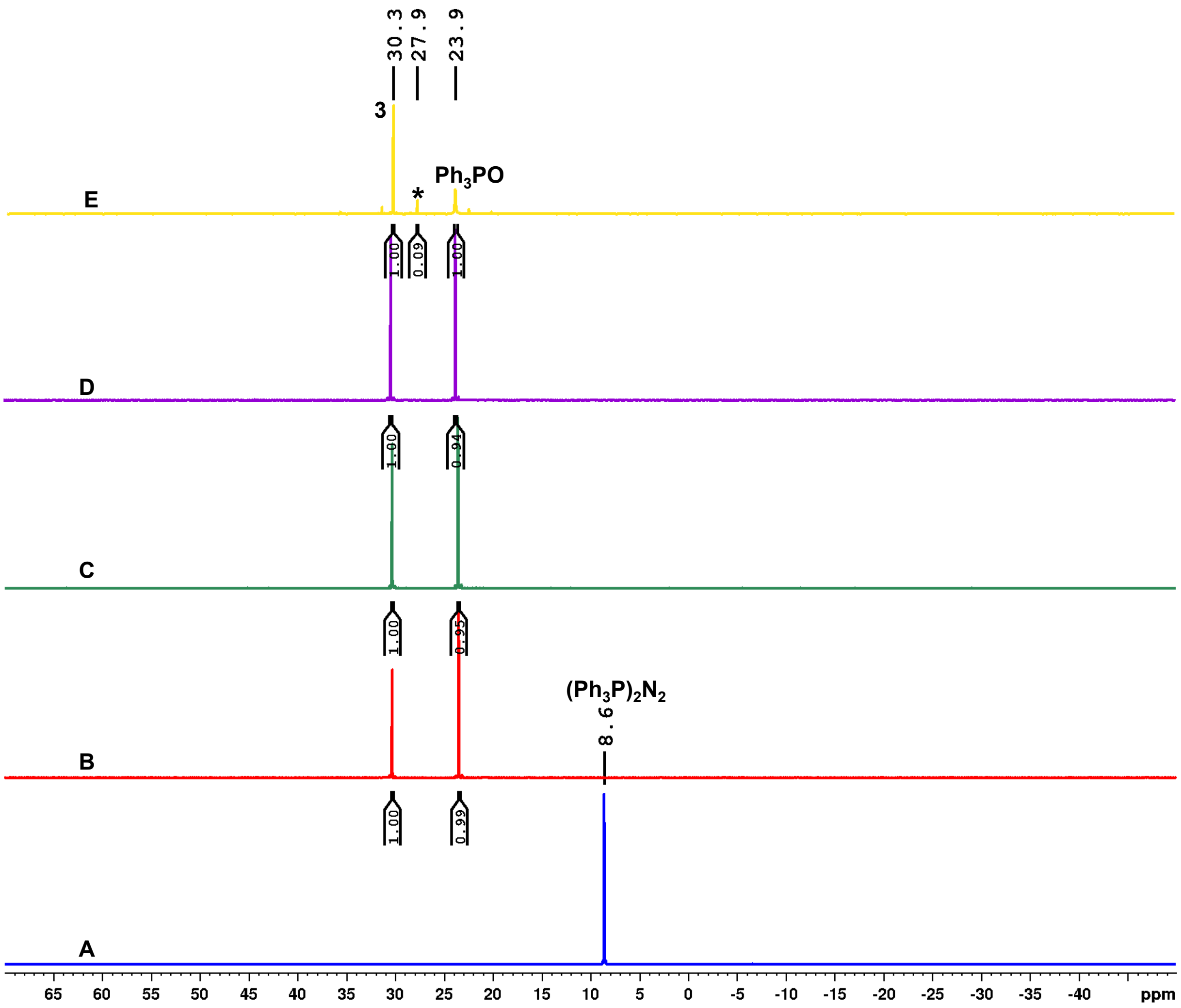

Figure S38. Stacked crude $^{31}P\{^1H\}$ NMR spectra (THF-d_8, 162.0 MHz, D1 = 30 s, * unidentified phosphorus containing species): **A**. $(Ph_3P)_2N_2$ in THF-d_8; **B**. After sonicating $W(CO)_6$ suspension for 10 min at RT; **C**. After sonication for 2 h at 60 °C; **D**. After heating to 80 °C for 2 h; **E**. After refluxing the reaction mixture at 100 °C for 2.5 h.

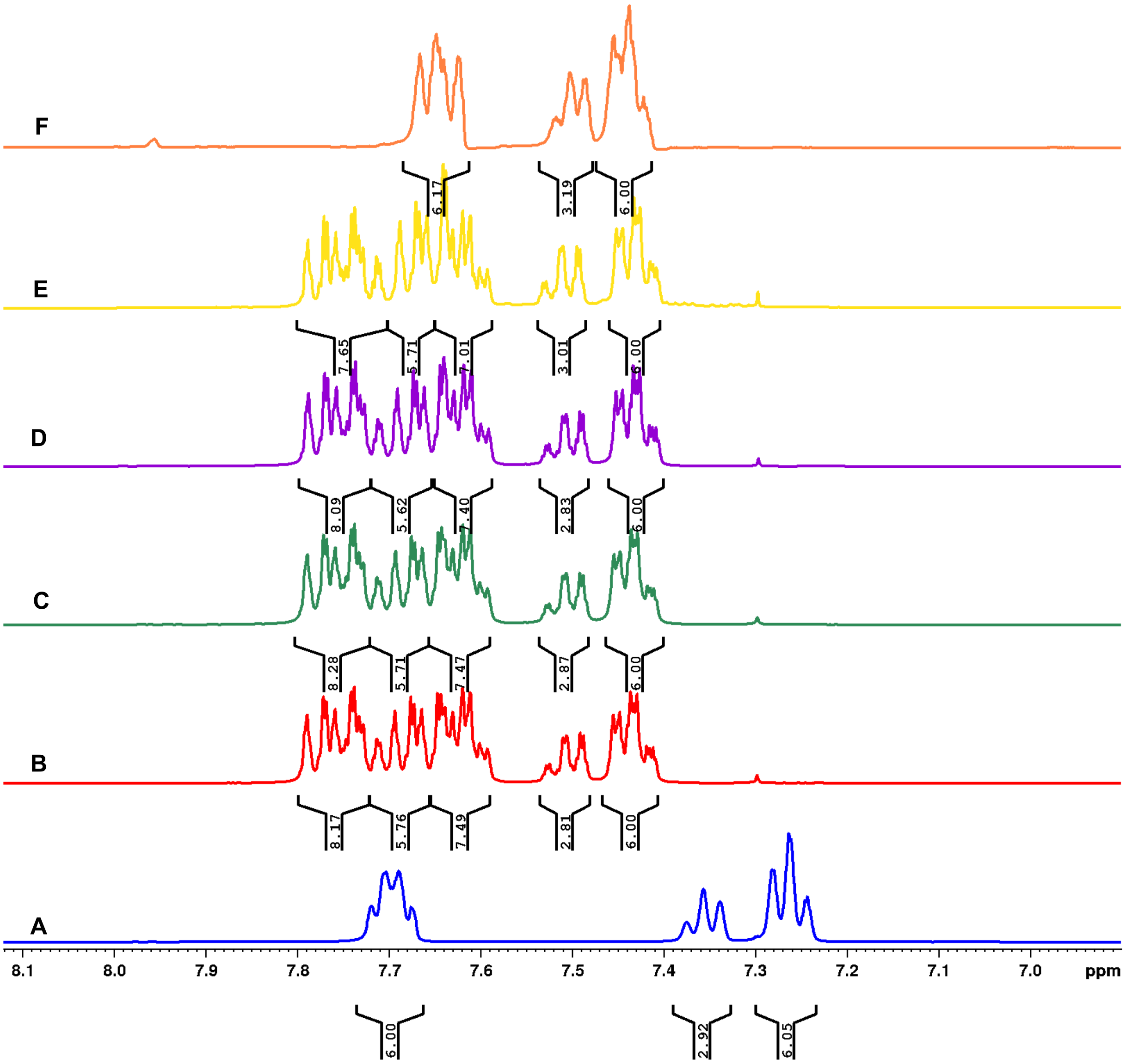

Figure S39. Stacked crude ^{1}H NMR spectra: **A**. [$(Ph_3P)_2N_2$] in THF-d_8; **B**. After suspended $W(CO)_6$, sonicated for 10 min at RT; **C**. After heating at 60 °C for 2 h; **D**. After heating at 80 °C for 2 h; **E**. After refluxing the reaction mixture at 100 °C for 2.5 h; **F**. reference spectrum of Ph_3PO; (THF-d_8, 400.0 MHz).

Reaction of [$(Ph_3P)_2N_2$] with [$Fe(CO)_5$] in *o*DFB or THF-d_8

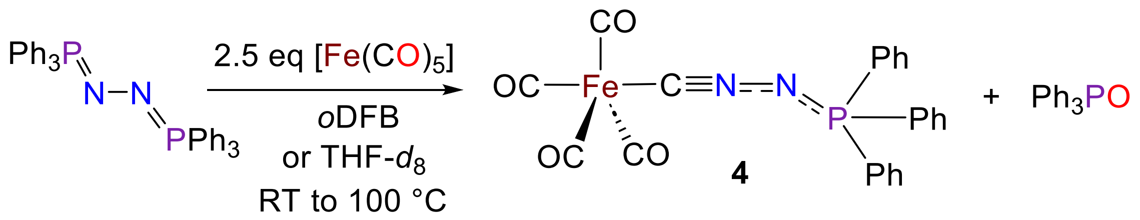

Inside the glovebox, red triphenylphosphineazine, [$(Ph_3P)_2N_2$] (30 mg, 0.05 mmol, 1.0 equiv.), was dissolved in 0.6 mL of either *o*DFB or THF-d_8 in separate *J*. Young NMR tubes. $Fe(CO)_5$ (27 mg, 0.14 mmol, 2.5 equiv., 18 µL) was added. The reaction mixture changed from red to yellow instantaneously. Analysis of the crude $^{31}P\{^1H\}$ NMR spectrum revealed the formation of [$(CO)_4FeCN_2PPh_3$] (**4**) and Ph_3PO in a 1:1 ratio.
Subsequently, the reaction mixture was sonicated at 60 °C for 2 h, affording a homogeneous yellow solution. The crude $^{31}P\{^1H\}$ NMR spectrum showed [$(CO)_4FeCN_2PPh_3$] (**4**) and Ph_3PO in an alleged 1:0.6 ratio, albeit under considerable line-broadening for Ph_3PO (Figures S40, 41). Line broadening / shifting of signals was also observed in the ^1H NMR spectroscopic analysis (Figure S42). Further heating to 80 °C for 2 h, followed by heating to 100 °C led to the disappearance of the Ph_3PO signal, yet not of [$(CO)_4FeCN_2PPh_3$] (**4**).

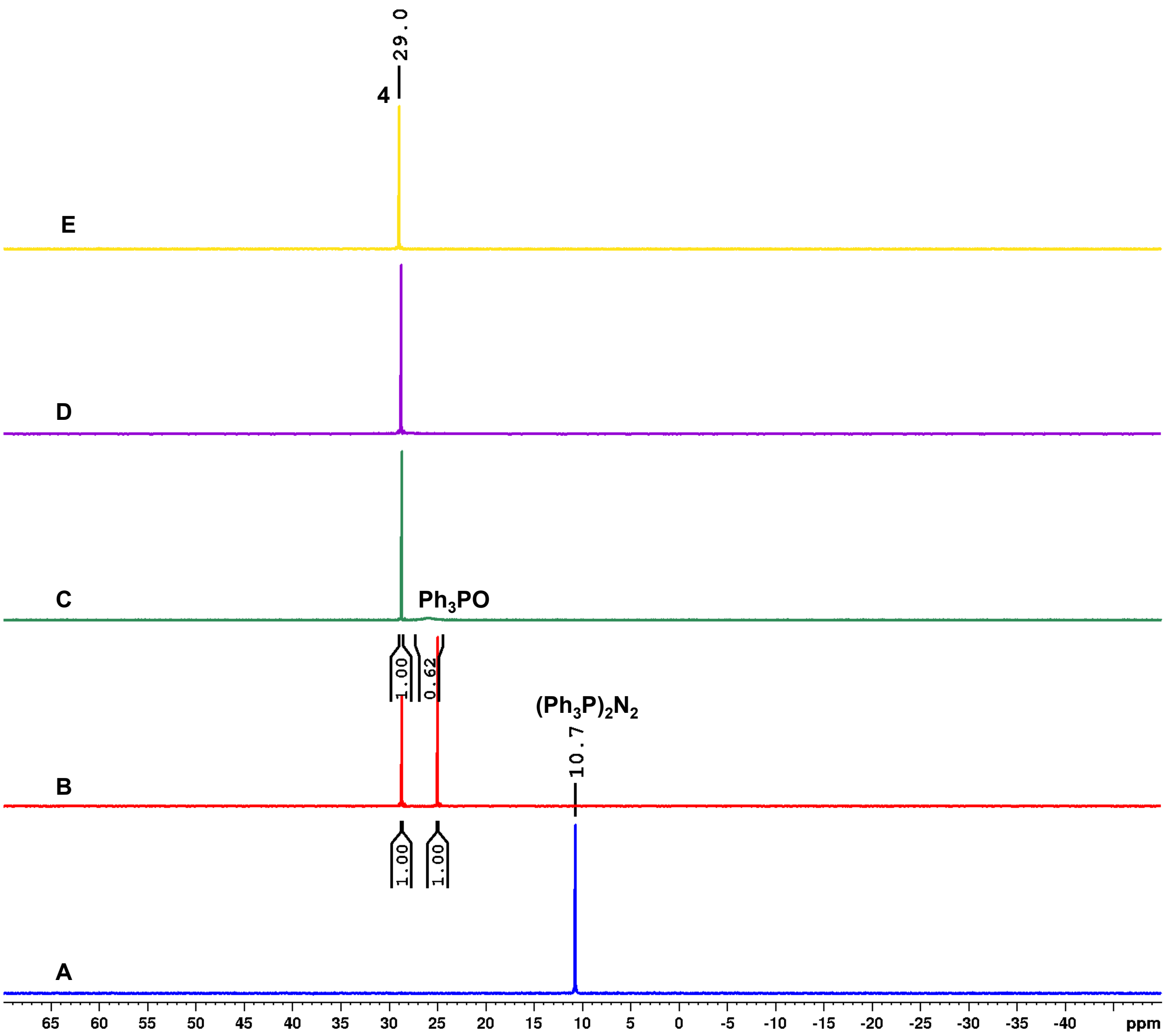

Figure S40. Stacked crude $^{31}P\{^1H\}$ NMR spectra (*o*DFB with DMSO-d_6, 162.0 MHz, D1 = 30 s): **A**. $(Ph_3P)_2N_2$ in *o*DFB; **B**. After mixing $Fe(CO)_5$ at RT; **C**. After heating at 60 °C for 2 h; **D**. After heating at 80 °C for 2 h; **E**. After refluxing the reaction mixture at 100 °C for 2.5 h.

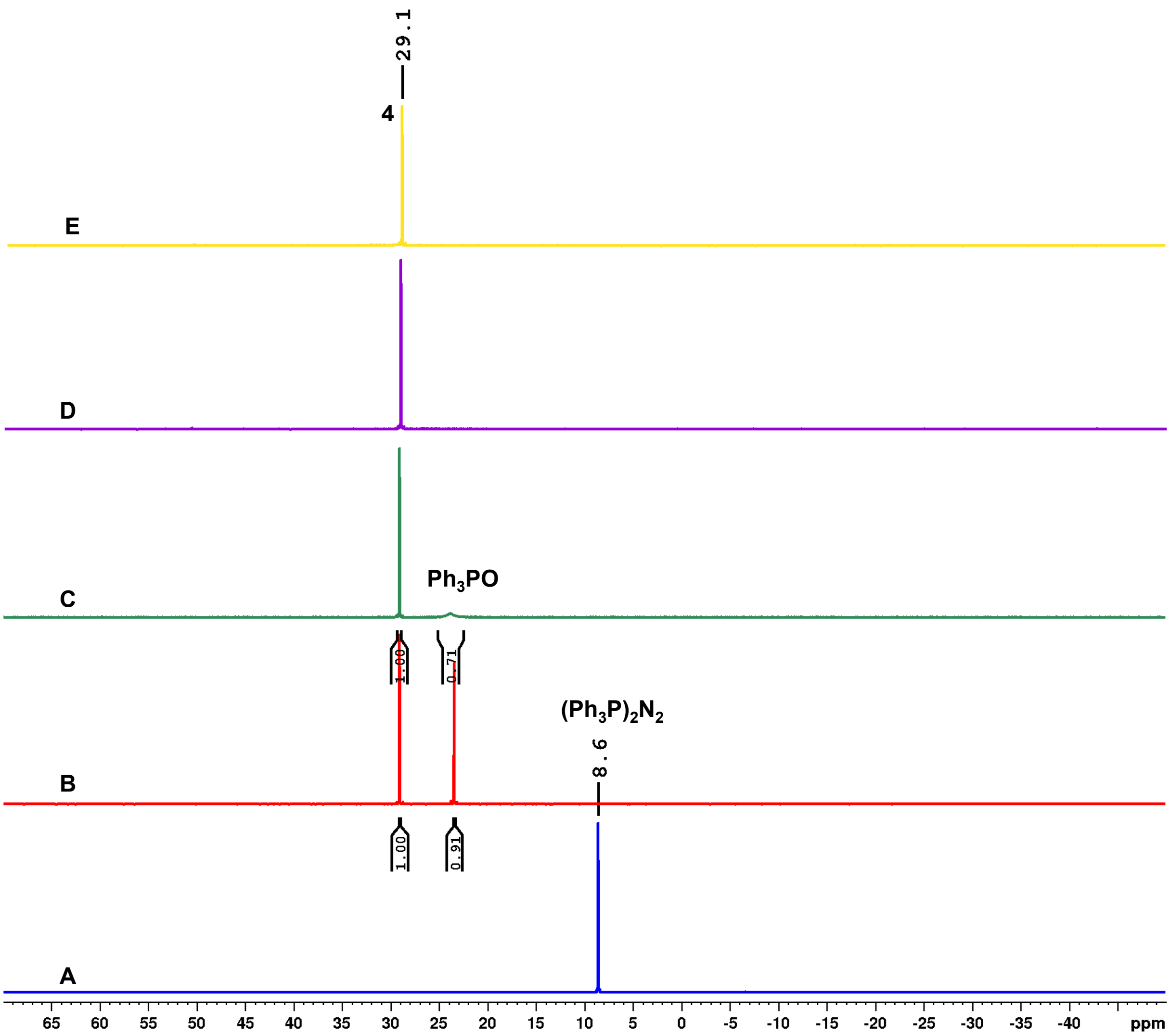

Figure S41. Stacked crude $^{31}P\{^{1}H\}$ NMR spectra: **A**. $[(Ph_3P)_2N_2]$ in THF-d_8; **B**. After mixing $Fe(CO)_5$ at RT; **C**. After heating to 60 °C for 2 h; **D**. After heating to 80 °C for 2 h; **E**. After refluxing the reaction mixture at 100 °C for 2.5 h; (THF-d_8, 162.0 MHz, D1 = 30 s).

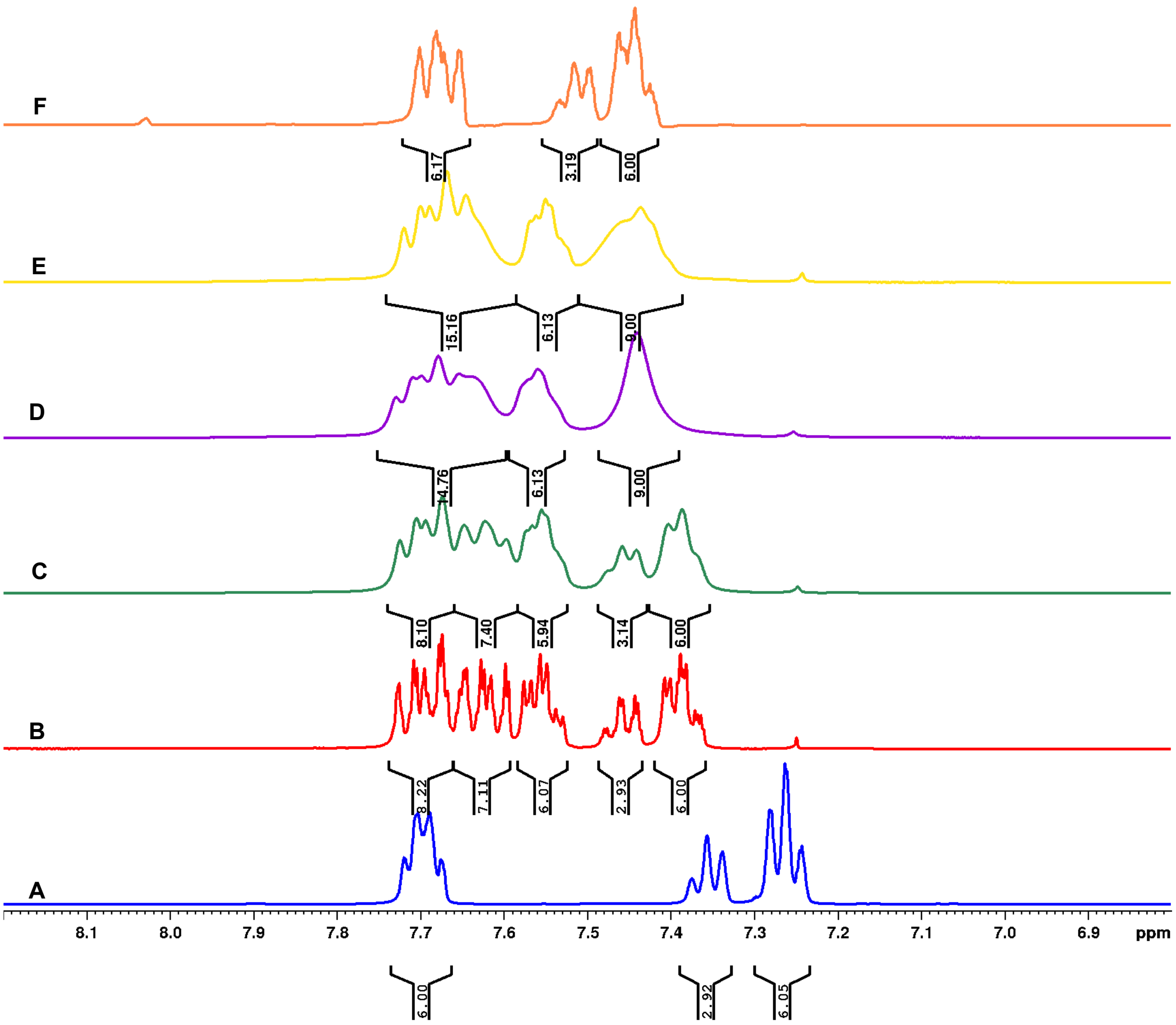

Figure S42. Stacked crude ^{1}H NMR spectra: **A**. $[(Ph_3P)_2N_2]$ in THF-d_8; **B**. After mixing $Fe(CO)_5$ at RT; **C**. After heating to 60 °C for 2 h; **D**. After heating at 80 °C for 2 h; **E**. After refluxing the reaction mixture at 100 °C for 2.5 h; **F**. reference spectrum of Ph_3PO; (THF-d_8, 400.0 MHz).

9 sc-XRD Structure Elucidation and Refinement

X-ray quality crystals were selected in Fomblin YR-1800 perfluoroether (Alfa Aesar) at ambient temperature. The sample was cooled, yet to different temperatures (please see Table S2 and special refinement details). The data set were collected using a Rigaku XtaLAB Synergy-S diffractometer with a microfocus sealed tube and a HyPix-6000HE Hybrid Photon Counting (HPC) detector (compound **2** and **4**) or a Bruker Apex II CCD diffractometer (compound **3**) using monochromated MoK_{α} (λ = 0.71073 Å) radiation. Data were collected at 133(2) (or 152(3), compound **3**) K and corrected for absorption effects using the multi-scan method. The structure was solved by direct methods using SHELXT[14] and was refined by full matrix least squares calculations on F^2 (SHELXL2019)[15] in the graphical user interface Shelxle.[16] Molecular structures represent the 50% probability level and have been drawn using Olex2.[17] A summary on standard crystallographic parameters as well as the CSD entry numbers within the Cambridge Crystallographic Data Centre (CCDC) is subsequently provided in Table S2.

Refinement Details

2: $(CO)_5MoCN_2PPh_3$; 3: $(CO)_5WCN_2PPh_3$; 4: $(CO)_4FeCN_2PPh_3$

All non H-atoms were located in the electron density maps and refined anisotropically. C-bound H atoms were placed in positions of optimized geometry and treated as riding atoms. Their isotropic displacement parameters were coupled to the corresponding carrier atoms by a factor of 1.2 (CH).

Molecular Structure Representation

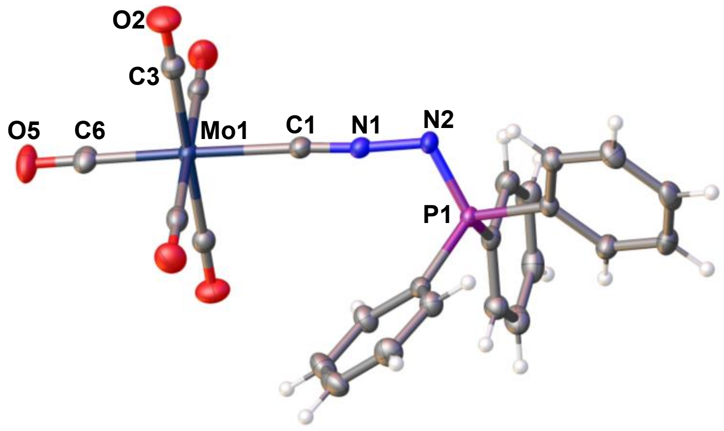

Figure S43. Molecular structure representation of **2**. Selected atom distances [Å] and angles [°]: Mo1-C6 2.0056(15), Mo1-C3 2.0612(16), Mo1-C1 2.1806(14), P1-N2 1.6262(13), O2-C3 1.1371(19), O5-C6 1.1456(19), N1-N2 1.3454(16), N1-C1 1.1555(19); C6-Mo1-C3 91.23(6), C3-Mo1-C1 90.97(6), N1-N2-P1 112.40(9), O2-C3-Mo1 178.89(14).

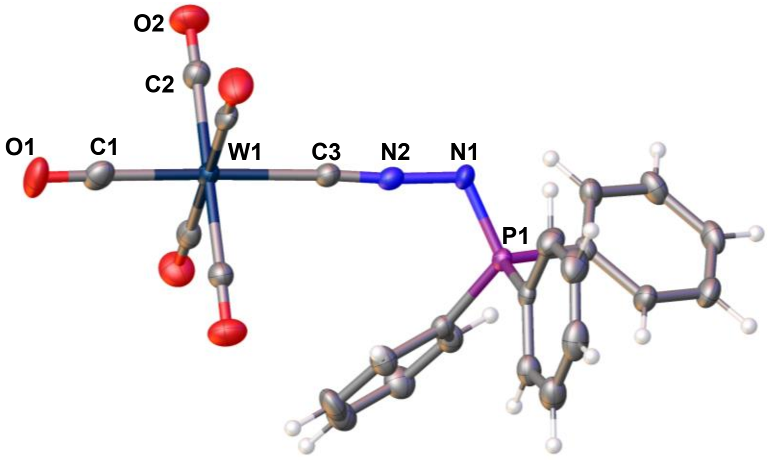

Figure S44. Molecular structure representation of **3**. Selected atom distances [Å] and angles [°]: W1-C1 1.997(4), W1-C2 2.050(4), W1-C3 2.168(4), P1-N1 1.620(3), O1-C1 1.153(5), O2-C2 1.139(5), N2-N1 1.346(4), N2-C3 1.145(5); C1-W1-C2 90.93(17), C1-W1-C3 177.81(16), C3-N2-N1 175.1(4), N2-N1-P1 112.8(3), O1-C1-W1 178.5(4), N2-C3-W1 177.0(3).

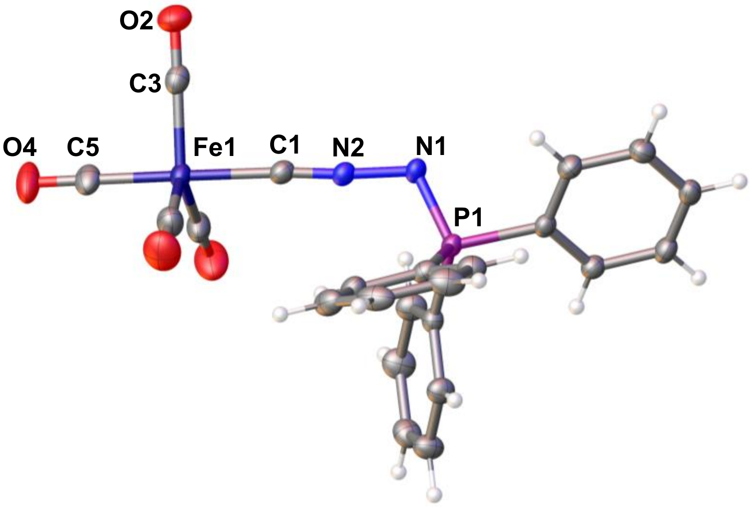

Figure S45. Molecular structure representation of **4**. Selected atom distances [Å] and angles [°]: Fe1-C5 1.7753(18), Fe1-C3 1.8008(17), Fe1-C1 1.9109(16), P1-N1 1.6315(13), N1-N2 1.3495(17), O2-C3 1.144(2), O4-C5 1.143(2), N2-C1 1.154(2); C5-Fe1-C3 91.14(8), N2-N1-P1 112.31(10), C1-N2-N1 175.20(15), N2-C1-Fe1 175.41(13), O2-C3-Fe1 179.13(16).

Table S2. Crystallographic details for compound **2**, **3**, and **4**.

**Compound**	**2**	**3**	**4**
Empirical formula	$C_{24}H_{15}N_2O_5PMo$	$C_{24}H_{15}N_2O_5PW$	$C_{23}H_{15}N_2O_4PFe$
Formula weight	538.29	626.20	470.19
Temperature/K	133(2) K	153(2)	133(2) K
Crystal system	Monoclinic	Monoclinic	Monoclinic
Space group	$P2_1/c$	$P2_1/c$	$P2_1/c$
a/Å	12.7993(3)	12.7762(5)	10.6633(3)
b/Å	15.3174(3)	15.3127(7)	10.9969(3)
c/Å	12.9190(3)	12.9092(6)	19.2319(6)
α/°	90	90	90
β/°	116.157(3)	116.1390(10)	103.435(3)
γ/°	90	90	90
Volume/Å^3	2273.41(10)	2267.24(17)	2193.48(11)
Z	4	4	4
ρ_{calc}g/cm^3	1.573	1.835	1.424
μ/mm^{-1}	0.686	5.204	0.792
F(000)	1080.0	1208.0	960.0
Crystal size/mm^3	0.240 × 0.200 × 0.080	0.17 × 0.12 × 0.03	0.20 × 0.10 × 0.06
Radiation	MoKα (λ = 0.71073)	MoKα (λ = 0.71073)	MoKα (λ = 0.71073)
2*Θ* range for data collection/°	4.406 to 54.198	3.55 to 56.616	4.356 to 54.216
Index ranges	-16 ≤h ≤ 16, -19 ≤ k ≤ 19, -16≤ l ≤ 16	-16 ≤ h ≤ 17, -20 ≤ k ≤ 20, -17 ≤ l ≤ 16	-13 ≤ h ≤ 13, -13 ≤ k ≤ 14, -24 ≤ l ≤ 24
Reflections collected	31975	35255	17064
Independent reflections	5024 [R_{int} = 0.0272]	5633 [R_{int} = 0.0592]	4838 [R_{int} = 0.0278]
Data/restraints/parameters	5024/0/298	5633/0/298	4838/0/280
Goodness-of-fit on F^2	1.049	1.028	1.066
Final R indexes [I>=2σ (I)]	R_1 = 0.0197, wR_2 = 0.0499	R_1 = 0.0288, wR_2 = 0.0612	R_1 = 0.0288, wR_2 = 0.0729
Final R indexes [all data]	R_1 = 0.0223, wR_2 = 0.0513	R_1 = 0.0454, wR_2 = 0.0672	R_1 = 0.0367, wR_2 = 0.0763
Largest diff. peak/hole /e Å^{-3}	0.365/-0.330	1.10/-0.70	0.405/-0.277
CCDC	2500618	2500685	2500617

10 References

1. Appel, R.; Schöllhorn, R. Triphenylphosphineazine $Ph_3P=N-N=PPh_3$. *Angew. Chem. Int. Ed. Engl.* **1964**, *3*, 805-805.
2. Holzmann, N.; Dange, D.; Jones, C.; Frenking, G. Dinitrogen as Double Lewis Acid: Structure and Bonding of Triphenylphosphinazine $N_2(PPh_3)_2$. *Angew. Chem. Int. Ed.* **2013**, *52*, 3004-3008.
3. Weinberger, B.; Fehlhammer, W. P. N-Isocyanoiminotriphenylphosphorane: Synthesis, Coordination Chemistry, and Reactions at the Metal. *Angew. Chem. Int. Ed. Engl.* **1980**, *19*, 480-481.
4. Stolzenberg, H.; Weinberger, B.; Fehlhammer, W. P.; Pühlhofer, F. G.; Weiss, R. Free and Metal-Coordinated (N-Isocyanimino)triphenylphosphorane: X-ray Structures and Selected Reactions. *Eur. J. Inorg. Chem.* **2005**, *2005*, 4263-4271.
5. Buchner, W.; Schenk, W. A. Carbon-13 NMR spectra of monosubstituted tungsten carbonyl complexes. NMR trans influence in octahedral tungsten (0) compounds. *Inorg. Chem.* **1984**, *23*, 132-137.
6. Cotton, F. A.; Musco, A.; Yagupsky, G. Vibrational spectra and bonding in metal carbonyls. V. New data for $XMn(CO)_5$ molecules and further examination of simplified force fields. *Inorg. Chem.* **1967**, *6*, 1357-1364.
7. Kettle, S. F. A.; Paul, I. The b_1 CO strectching mode in the infrared spectra of C_{4v} $M(CO)_5X$ complexes. *Inorg. Chim. Acta* **1968**, *2*, 15-16.
8. Weinberger, B.; Fehlhammer, W. P. Metallkomplexe funktioneller Isocyanide, VIII. (Isocyanimino)triphenylphosphoran-Metallkomplexe. *Chem. Ber.* **1985**, *118*, 42-50.
9. Cook, J. B.; Nicholson, B. K.; Smith, D. W. A structural, spectroscopic and theoretical study of the triphenylphosphine chalcogenide complexes of tungsten carbonyl, $[W(XPPh_3)(CO)_5]$, X=O, S, Se. *J. Organomet. Chem.* **2004**, *689*, 860-869.
10. Darensbourg, D. J.; Pala, M.; Simmons, D.; Rheingold, A. L. Chemical and structural characterization of $W(CO)_5OPPh_2NPPh_3$. A novel tungsten carbonyl complex containing a phosphine oxide ligand derived from the bis(triphenylphosphine)nitrogen(1^+) cation. *Inorg. Chem.* **1986**, *25*, 3537-3541.
11. Bio, M. M.; Javadi, G.; Song, Z. J. An improved synthesis of N-isocyanoiminotriphenylphosphorane and its use in the preparation of diazoketones. *Synthesis* **2005**, *2005*, 19-21.
12. Ruppert, I.; Appel, R. Ein neues Verfahren zur Darstellung von N-Cyaniminophosphoranen. *Chem. Ber.* **1978**, *111*, 751-758.
13. Sun, H.; DiMagno, S. G. Anhydrous Tetrabutylammonium Fluoride. *J. Am. Chem. Soc.* **2005**, *127*, 2050-2051.
14. Sheldrick, G. SHELXT - Integrated space-group and crystal-structure determination. *Acta Crystallogr., Sect. A: Found. Adv.* **2015**, *71*, 3-8.

15. Sheldrick, G. Crystal structure refinement with SHELXL. *Acta Crystallogr., Sect. C: Struct. Chem.* **2015**, *71*, 3-8.
16. Hubschle, C. B.; Sheldrick, G. M.; Dittrich, B. ShelXle: a Qt graphical user interface for SHELXL. *J. Appl. Crystallogr.* **2011**, *44*, 1281-1284.
17. Dolomanov, O. V.; Bourhis, L. J.; Gildea, R. J.; Howard, J. A. K.; Puschmann, H. OLEX2: a complete structure solution, refinement and analysis program. *J. Appl. Crystallogr.* **2009**, *42*, 339-341.
